# Selecting for Altered Substrate Specificity Reveals the Evolutionary Flexibilityof ATP-Binding Cassette Transporters

**DOI:** 10.1016/j.cub.2020.02.077

**Published:** 2020-03-26

**Authors:** Sriram Srikant, Rachelle Gaudet, Andrew W. Murray

**Affiliations:** 1 Department of Molecular and Cellular Biology, Harvard University, 52 Oxford Street, Cambridge, MA 02138, USA; 2 Present address: Department of Biology, MIT, Cambridge, MA 02139, USA; 3 Lead Contact

## Abstract

ATP-binding cassette (ABC) transporters are the largest family of ATP-hydrolyzing transporters, which import or export substrates across membranes, and have members in every sequenced genome. Structural studies and biochemistry highlight the contrast between the global structural similarity of homologous transporters and the enormous diversity of their substrates. How do ABC transporters evolve to carry such diverse molecules and what variations in their amino acid sequence alter their substrate selectivity? We mutagenized the transmembrane domains of a conserved fungal ABC transporter that exports a mating pheromone and selected for mutants that export a non-cognate pheromone. Mutations that alter export selectivity cover a region that is larger than expected for a localized substrate-binding site. Individual selected clones have multiple mutations, which have broadly additive contributions to specific transport activity. Our results suggest that multiple positions influence substrate selectivity, leading to alternative evolutionary paths toward selectivity for particular substrates and explaining the number and diversity of ABC transporters.

## In Brief

Srikant et al. find that mutations at many different positions in an ABC transporter of fungal mating pheromone have roughly additive effects on substrate recognition. This helps explain the evolvability of ABC transporters to transport a remarkable variety of substrates and their presence as the largest protein family across all domains of life.

## INTRODUCTION

During evolution, many genes have duplicated and diverged to acquire new functions. ATP-binding cassette (ABC) transporters, the largest single family of proteins in the Protein Families (PFAM) database, are an excellent example of protein diver-sification. Clearly, orthologous proteins transport different substrates, and most genomes contain paralogous transporters that either import or export different substrates, playing different physiological roles. ABC transporters contain cytoplasmic nucleotide-binding domains (NBDs) with conserved motifs for binding and hydrolyzing ATP, connected to transmembrane domains (TMDs) that undergo conformational changes to transport substrates across membranes [1, 2].

Many eukaryotic and prokaryotic homologs belong to the type I ABC exporter subfamily, whose members export substrates with a wide variety of physicochemical properties. For example, MsbA transports a large glycosylated lipid (lipopolysaccharide [LPS]) for bacterial outer membrane biogenesis [3, 4], and the transporter for antigen processing (TAP) exports the peptides that are presented to the adaptive immune system of vertebrates from the cytosol to the endoplasmic reticulum [5]. The substrate selectivity of ABC exporters determines the multidrug resistance phenotype of cancers (e.g., P-glycoprotein or P-gp) [6] and parasitic pathogens (*P. falciparum* MDR1) [7]. Sequence conservation in the TMDs of homologous exporters has not been helpful in identifying a conserved binding site for cognate substrates in orthologous or paralogous exporters. ABC transporters oscillate between two states: one with a central substrate-binding cavity exposed to the cytoplasm (inward-open) and one with roughly the same surface exposed to the other side of the membrane (outward-open) [2]. Structures of inward-open exporters with different substrates are remarkably similar, and substrate is expected to bind over the large, chemically heterogeneous surface of the TMD cavity [2]. Biochemical crosslinking and mutagenesis experiments on P-gp and TAP have identified positions in the TMD cavity involved in substrate recognition [8–10], but a system to characterize the sequence determinants of substrate selectivity in type I ABC exporters has not been reported.

**Figure 1.**
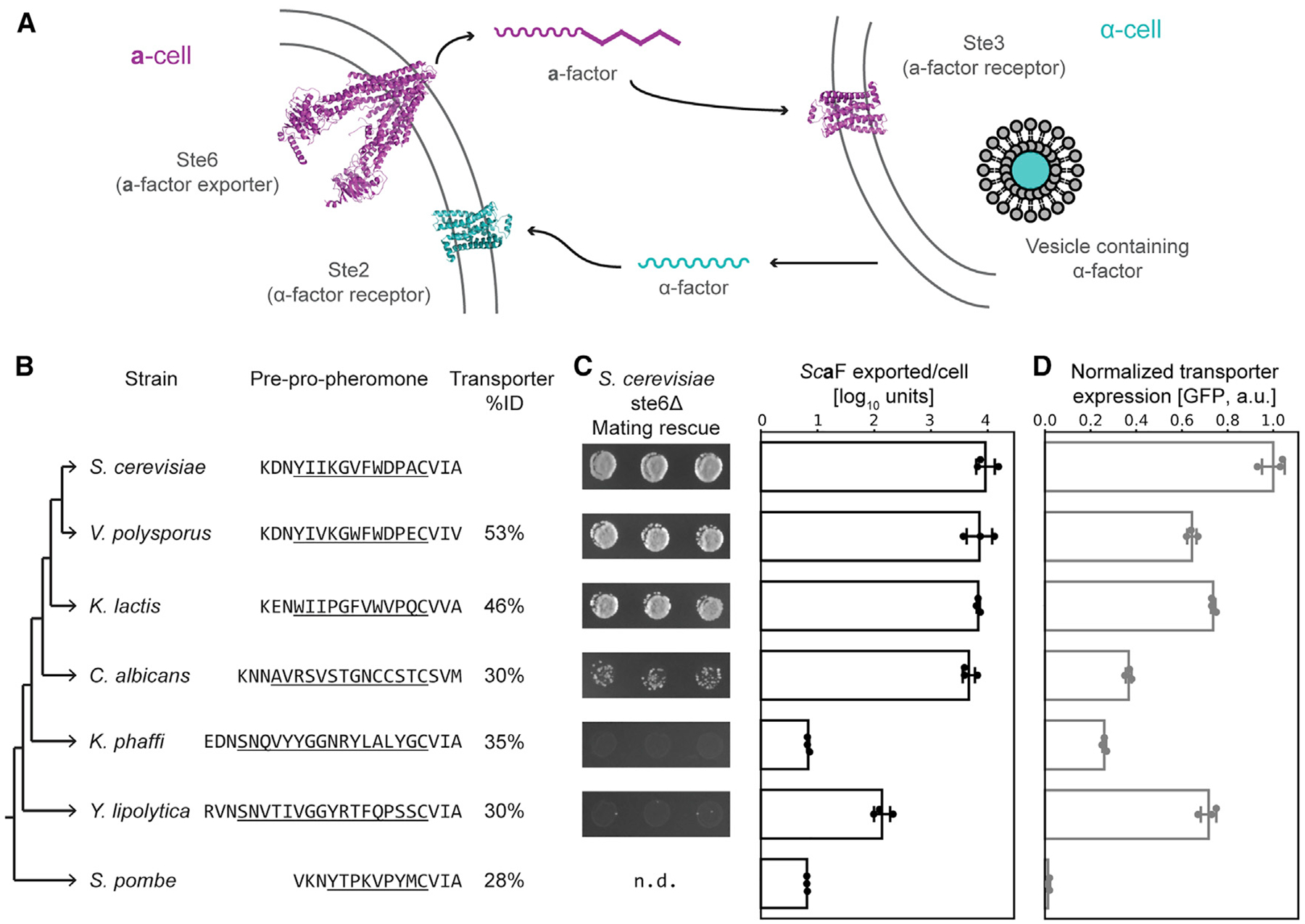
Homologous Pheromone Transporters Can Partially Rescue a-Factor Export from S. cerevisiae (A) Mating in fungi is initiated by a two-way pheromone communication between haploids of different mating types. In *S. cerevisiae*, a-cells secrete the 13-amino-acid peptide *α*-factor (*α* F) via secretory vesicles; *α* F is recognized by a G-protein coupled receptor (GPCR), Ste2, on a-cells. The farnesylated 12-amino-acid a-factor is made in the cytoplasm of a-cells; exported by a dedicated ABC exporter, Ste6; and recognized by a different GPCR, Ste3, on *α* -cells. (B) A cladogram of the yeasts used in this study, highlighting the sequence of a-factor-like pheromones (mature peptides are underlined and only a portion of the N-terminal region of the initial peptide is shown) [11, 19–21], and the % identity of orthologous pheromone transporters to *S. cerevisiae* Ste6 is shown. (C) Bioassays to measure *Sc*a-factor export from *ste6Δ S. cerevisiae* a-cells that express orthologous pheromone transporters (the transporter sources are the species aligned in B). Left: *Sc*a-factor export measured indirectly via mating rescue is shown (n.d. is not determined). Right: *Sc*a-factor export measured by purifying a factor from culture and quantifying its ability to arrest *α* -cells (see STAR Methods for details). *Sc*aF exported/cell is the log_10_ value of the fraction of dilution factor of extract that can still arrest *α* -cell growth to the number of a-cells used to collect the extract. Raw data from biological triplicates are plotted as dots with average plotted as bar graphs with standard deviations. (D) The expression of Ste6 homologs, measured by flow cytometry as the median fluorescent signal of cells expressing GFP-tagged transporters (~30,000 events). Normalized fluorescence (empty plasmid control set to 0 and *Sc*Ste6 set to 1) data from biological triplicates are plotted as dots with average plotted as bar graphs with standard deviations. See also Figure S1.

To study substrate selectivity in ABC exporters, we took advantage of the pheromones used for fungal mating. In the species-rich group of fungi (Dikarya), mating depends on the exchange of diffusible peptide pheromones between the two haploid mating partners (Figure 1A). In the budding yeast,*S. cerevisiae*, a farnesylated pheromone, a-factor [11, 12], is synthesized in the cytosol of *MAT*a haploid cells (a-cells) and exported into the medium by a type I ABC exporter, Ste6 [13]. The restriction of Ste6’s function and expression to a-cells [14, 15] and biochemical assays of a-factor transport [16, 17] strongly argue for Ste6 being a dedicated a-factor (farnesylated pheromone) exporter. With the known variation in the peptide sequence of farnesylated pheromones across fungal phylogeny [18], we expect coevolution of substrate selectivity in the homologous pheromone exporters. Because the physiologically relevant substrates evolve across the Dikarya, we hypothesized that the Ste6 family of pheromone exporters would coevolve with the pheromones, allowing us to study the variation of substrate selectivity.

We started by testing the ability of Ste6 homologs from different Ascomycetes to rescue *S. cerevisiae* a-factor export. The Ste6 homolog from *Y. lipolytica*, which last shared a common ancestor with *S. cerevisiae* 320 mya, exports *S. cerevisiae* a-factor poorly but exports the *Y. lipolytica* a-factor, which lacks any sequence similarity to *S. cerevisiae* a-factor [22]. This functional difference in substrate transport allowed us to develop a high-throughput assay for mutations in a transporter that increased its ability to export a-factor from *S. cerevisiae*. Using libraries of mutagenized *Y. lipolytica* a-factor transporters, we identified regions of the TMD that affect substrate selectivity by allowing transport of this “novel” substrate. We hypothesize that the large target size for mutations that improve a-factor export creates many alternative paths for the evolution of paralogous exporters and explains the success of the ABC transporter family in evolving to “solve” the problem of transporting different substrates.

## RESULTS

### The Pheromone and Transporter Form a Conserved Module in the Mating Systems of Ascomycetes

Pheromones of many yeasts (unicellular fungi) have been identified [11, 19–21], and their peptide sequences vary across phylogeny. In one mating type, these pheromones are small peptides that are C-terminally farnesylated and methyl esterified (Figures 1A and S1A); they undergo maturation through a conserved set of enzymes, highlighting the ancestral role of farnesylated pheromones in fungal mating [23]. We used these common features and a bioinformatic approach to identify and experimentally validate the farnesylated pheromone from*Y. lipolytica* [22]. Orthologs of Ste6 (ABCB exporters) [24–26] can be reliably identified by sequence homology (Figure 1B). To test the function of homologous pheromone transporters from fungi, we started with seven yeast species whose mating systems have been studied and are estimated to have last shared a common ancestor roughly 320 million years ago (mya) [27].

In *S. cerevisiae*, Ste6 is expressed only in *MAT*a-cells, and *ste6Δ* cells are mating deficient [14], providing a biological assay for a-factor transport. We expressed Ste6 orthologs from other species in *ste6Δ S. cerevisiae MAT*a-cells and used a mating rescue experiment to show that orthologous transporters have functionally diverged (Figures 1C and 1D). The efficiency of mating roughly correlates with the phylogenetic distance between *S. cerevisiae* and the yeast containing the supplied Ste6 ortholog, which is consistent with coevolution of the transporter with the pheromone it transports (Figure 1B). To confirm that the efficiency of mating is a good measure of transporter function, we assayed pheromone export directly by collecting exported a-factor from growing cultures and using a serial dilution bioassay to measure the quantity of exported pheromone (Figure 1C). We also measured the expression and localization of Ste6 orthologs by tagging them with GFP (Figures 1D and S1B). A Ste6 ortholog that was strongly expressed but strongly deficient in a-factor export is an ideal substrate to mutate and select for increased export of *S. cerevisiae* a-factor. Because the *Y. lipolytica* Ste6 (*Yl*Ste6) and *S. cerevisiae* Ste6 (*Sc*Ste6) are equally strongly expressed and show a 100-fold difference in transport of *Sc*a-factor, we set out to identify mutations in *Yl*Ste6 that would increase the transport of *Sc*a-factor and shed light on the evolution of substrate specificity in the pheromone transporter family. By starting with a distant homolog that had weak but detectable exporter activity for *Sc*a-factor, we hypothesized that it should be possible to select for mutations that allow it to recognize a substrate that differs in length, sequence, and amino acid composition from the pheromone it normally transports. *Yl*Ste6 is only 31% identical in amino acid sequence to *Sc*Ste6, which is only slightly more homologous than the 26% identity between *Sc*Ste6 and human P-glycoprotein.

### Building a Selection System for a-Factor Transport

**Figure 2.**
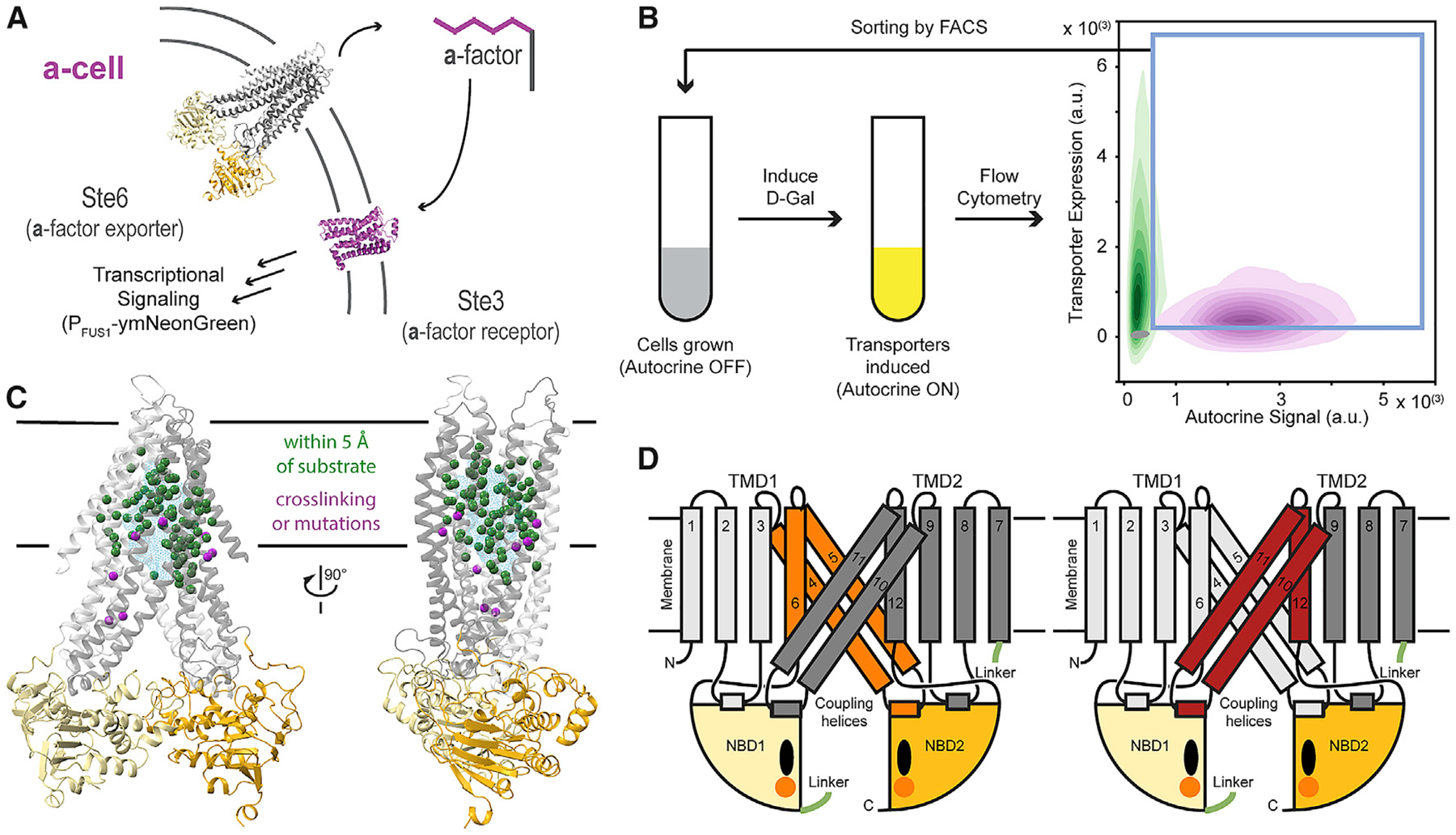
Two-Color Autocrine System Allows for Selection of Cells Expressing Transporters Functional in the Export of S. cerevisiae a-Factor (A) An autocrine system was designed in a-cells by inducibly expressing the a-factor-sensitive GPCR (P_GAL1_-STE3) and a-factor transporter (P_GAL1_-STE6) and including a transcriptional reporter for pheromone stimulation (P_FUS1_-*mNeonGreen*) [28]. (B) Schematic of a round of enrichment for increased a-factor secretion. A period of replicative growth with the autocrine system OFF is followed by FACS after turning the autocrine reporter ON (indicated by gray to yellow change). Cells were gated on both transporter expression and autocrine signal (blue box) and sorted into autocrine OFF media for expansion before the next round of selection. The 2D histogram shows the flow cytometry signal for populations of cells expressing *Sc*Ste6 (magenta), *Yl*Ste6 (green), and autocrine OFF *Yl*Ste6 (gray). Populations (n > 25,000 events) are plotted as density contours of autocrine reporter signal and transporter expression signal. (C) Model of *Yl*Ste6 (in its inward-open conformation) highlighting positions proximal to substrate density (C*α* as green spheres) in structures of three ABC proteins (P-gp bound to Zosuquidar, PDB: 6FN4; P-gp bound to QZ-Ala, PDB: 4Q9I; MsbA bound to LPS, PDB: 5TV4; and MRP1 bound to Leukotriene C4, PDB: 5UJA) and positions homologous to residues in TAP and P-gp that have been shown to affect substrate recognition by crosslinking, allelic variants, and constructed mutations (magenta spheres) [2]. The cyan mesh shows the cumulative density of substrates listed above. (D) Schematic of *Yl*Ste6 to illustrate the contiguous TM4–6 (orange, left) and TM10–12 (brick red, right) regions chosen for library construction. These TMs contain many positions highlighted in the model in (C) and combine to form the cytoplasm-facing cavity in the inward-open state. TMD1 and TMD2 are shown in grays, NBD1 and NBD2 in yellows, nucleotide as black ovals, and Mg^2+^ as orange spheres. See also Figures S1 and S2.

We used a genetic selection to find mutations that increase transport function because it allows us to start with a large, unbiased library of variants. Figure 2A shows the design of our selection strain: expressing the a-factor receptor, Ste3, in a-cells with a pheromone-stimulated reporter (P_FUS1_-ymNeonGreen) creates a cell whose response to the pheromone it exports can be measured via whole-cell fluorescence (see STAR Methods). By tagging the transporter with an orthogonal fluorescent protein (ymKate2), we created a two-color autocrine system that simultaneously reports on the expression of the transporter and its ability to export *Sc*a-factor. We tested this system by assaying cells expressing either *Sc*Ste6 or *Yl*Ste6 with flow cytometry to confirm that *Yl*Ste6 was defective in *Sc*a-factor export, and transporters with differing abilities to export *Sc*a-factor can be distinguished using the autocrine signal they produce (Figures 2B and S2A).

A combination of genetics [8], structural analysis [2], and biochemistry [10] highlights the relevance of transmembrane (TM) helices 4–6 and 10–12, which line the TMD cavity, for substrate selectivity (Figures 2C and 2D). Substrate-interacting residues in transporter structures (TAP, P-gp, MRP1, and MsbA) form large interaction surfaces with physicochemical properties that match the cognate substrate [2]. Given the size of the pheromone substrates and a large substrate interaction surface, we expected that multiple mutations might be needed to alter the substrate selectivity of Ste6. Therefore, rather than systematically exploring the effect of all single mutations in this region [29, 30], we used random mutagenesis, via error-prone PCR, to build mutant libraries (each with 10^4^ to 10^5^ members) on a yeast-replicating plasmid, with each clone containing multiple mutations (Figure S2B). One set of libraries mutated TMs4–6 (residues 199–359) and the other TMs10–12 (residues 851–1,012). Together, these two regions constitute the pseudo-symmetric sets of TMs that form a large part of the TMD cavity surface (Figure 2D).

We ran multiple mutated libraries of *Yl*Ste6 through a selection using our autocrine system to enrich for mutants in *Yl*Ste6 that improve a-factor export (STAR Methods; Figures S2C and S2D). In summary, the fluorescence-activated cell sorting (FACS)-based selection allowed us to enrich a library of mutant transporters for increased activity and isolate and confirm independent clones from the enriched population, and therefore connect a transport phenotype to specific clones.

### Sequencing Identifies Mutations across the TMD in Selected Clones

**Figure 3.**
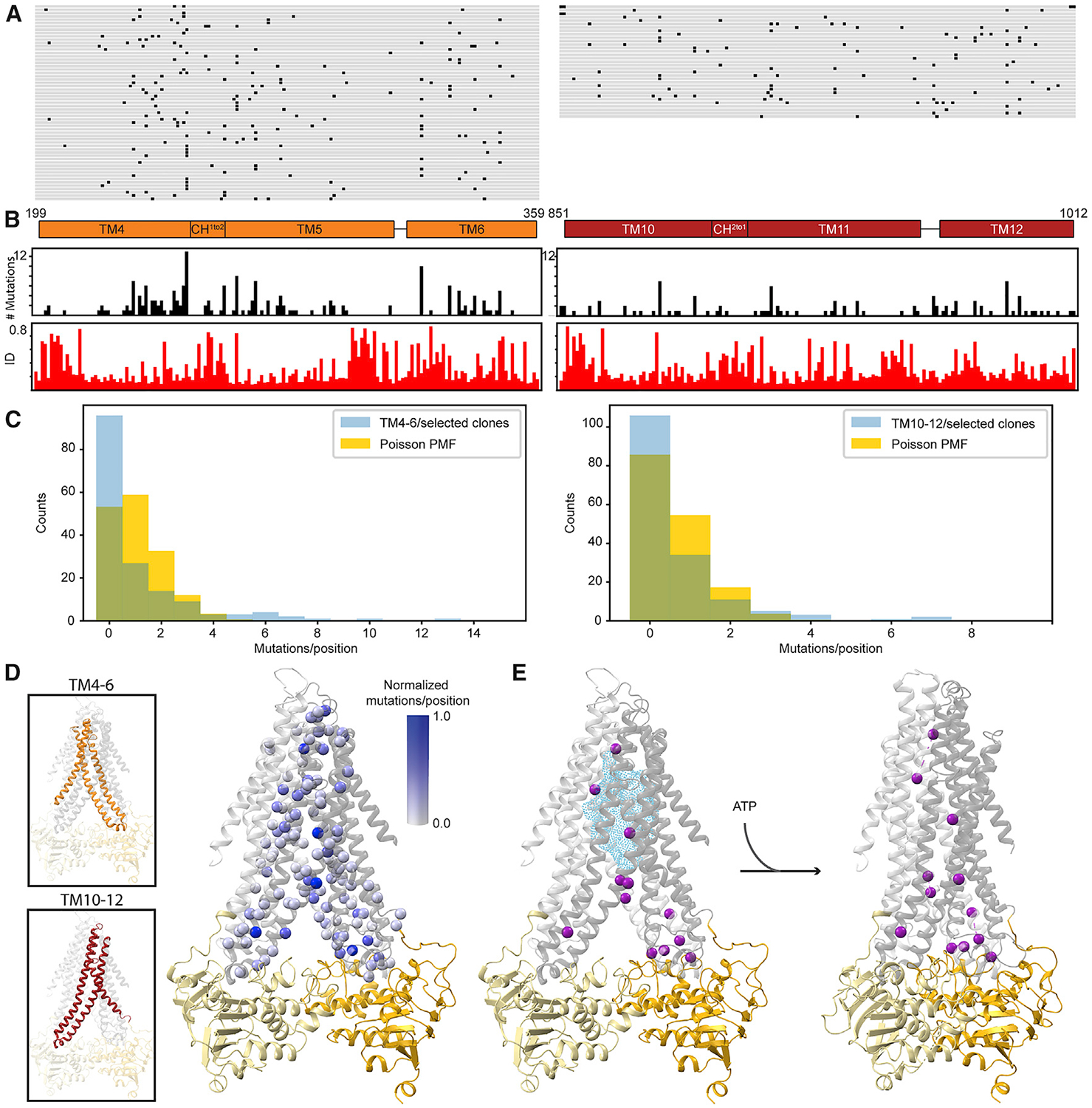
Mutations across the Entire Mutagenized Region Can Increase Autocrine Signaling (A) Plasmids that conferred increased autocrine signaling from the TM4–6 and TM10–12 libraries were sequenced, and the translated regions of interest are aligned to WT *Yl*Ste6 with positions of non-synonymous mutations marked in black for each clone. On the left are the 59 unique clones (90 sequenced total) selected from TM4–6 libraries and on the right are the 33 unique clones (153 sequenced total) selected from TMs10–12 libraries. (B) The number of mutations at each position summed over all unique selected clones is plotted in black bar plots for TM4–6 (left) and TM10–12 (right). The fractional mean pairwise sequence identity of residues in aligned fungal Ste6 sequences across the mutagenized regions TM4–6 (left) and TM10–12 (right) calculated from an alignment of 1,126 fungal pheromone exporters are plotted in red bar plots. The TM boundaries are schematized in orange (TMs 4–6 and coupling helix CH^1to2^; residues 199–359) and red (TMs 10–12 and coupling helix CH^2to1^; residues 851–1,012) above the bar plots. (C) Histogram of number of mutations per position summed over all unique selected clones from TM4–6 (left) and TM10–12 (right) libraries. The histogram of the mutations/positions (blue) has a longer tail than a Poisson probability mass function (PMF) distribution of the same number of mutations (gold). Chi-square (*X*^*2*^) goodness-of-fit test statistic for TM4–6 is 3.2 3 10^7^ (p ~ 0) and for TM10–12 is 5.7 × 10^3^ (p ~ 0) against a Poisson distribution of the same number of mutations. (D) The normalized (relative to the frequency of the most mutated position in each library) number of mutations per position is mapped on the inward-open homology model of *Yl*Ste6 with the corresponding C*α* spheres colored white to blue proportional to the values plotted in (B). The insets highlight the symmetric regions TM4–6 and TM10–12 that are mutated in our libraries. (E) Statistically enriched positions in our selected clones (magenta C*α* spheres) are mapped on the inward-open (left) and outward-open (right) homology models of *Yl*Ste6. The positions were identified by considering a Poisson distribution with same mean as our observed data (C) as a null expectation. Our enrichment threshold is that the observed mutations-per-position value is 10-fold higher than that predicted by the Poisson distribution. The thresholds are ≥6 for TM4–6 clones and ≥5 for TM10–12 clones, yielding 12 positions (9 from TM4–6 and 3 from TM10–12). The cyan cloud represents cumulative substrate density from aligned substrate-bound structures of P-gp, MsbA, and MRP1 as in Figure 2C. See also Figure S3 and Mendeley Source Data File 1.

We analyzed the *Yl*Ste6 mutants that had an improved autocrine signal and presumably have increased *Sc*a-factor transport. We isolated and sequenced a total of 243 “top” clones (of about 1,500 tested clones with increased autocrine signal relative to wild-type [WT] *Yl*Ste6) (Figure S3A) from the enriched populations. We confirmed that increased autocrine signal can be attributed to the plasmids containing mutated transporters by isolating plasmids and transforming them into naive cells (Figure S3B). We used Sanger sequencing to identify the mutations present in each clone. Sequencing the 243 clones that give high autocrine signals identified 92 unique clones (59 clones from TM4–6 and 33 clones from TM10–12), suggesting that we found most of the clones in our libraries that strongly increased autocrine signaling. Multiple mutations are present in most clones with an increased autocrine signal (Figure S3C). Clones containing a comparable number of mutations were well represented in the starting libraries (Figure S2B). Rather than a few hotspots composed of specific positions or small regions that might represent a specific substrate contact, we observe mutations distributed across the entire mutated region in the enriched clones (Figures 3A and 3B). We also plotted the change in autocrine signal (subtracting the signal from WT *Yl*Ste6) versus the number of mutations contained in the 59 TM4–6 and 33 TM10–12 selected clones (Figure S3D). There is a positive correlation between the autocrine signal and mutation count for the selected clones, stronger for TM4–6 than TM10–12 clones, particularly when considering bins with greater than five clones, i.e., up to four mutations per clone (Pearson coefficients 0.364 and 0.191 for TMs4–6 and TMs10–12, respectively). This suggests that accumulating mutations can increase autocrine signal.

We compared the resulting distributions of the number of mutations per position to the expectation that the mutations were distributed randomly across the mutated regions in a Poisson process (Figure 3C). Relative to this expectation, there are more positions with zero mutations, suggesting that some conserved positions cannot be mutated without severely inhibiting transporter activity. In contrast, twelve positions have been mutated many times more often than expected, suggesting that they are likely to affect substrate selectivity (Figure 3). We also sequenced clones from a control population enriched by gating on transporter expression rather than the autocrine reporter (Figure S3E). Examining transporter activity in these clones confirmed that many mutations do not increase the autocrine signal (Figure S3E), demonstrating that our selection enriches for mutations that alter the substrate selectivity and increase the transport of *Sc*a-factor by the transporter.

Plotting the number of mutations per position from the clones selected for increased autocrine signaling on homology models shows that mutations in selected clones are distributed throughout and beyond the TMD cavity surface (Figure 3D), as are the 12 statistically enriched positions (Figure 3E).

### Selected Clones Have Increased Pheromone Export

**Figure 4.**
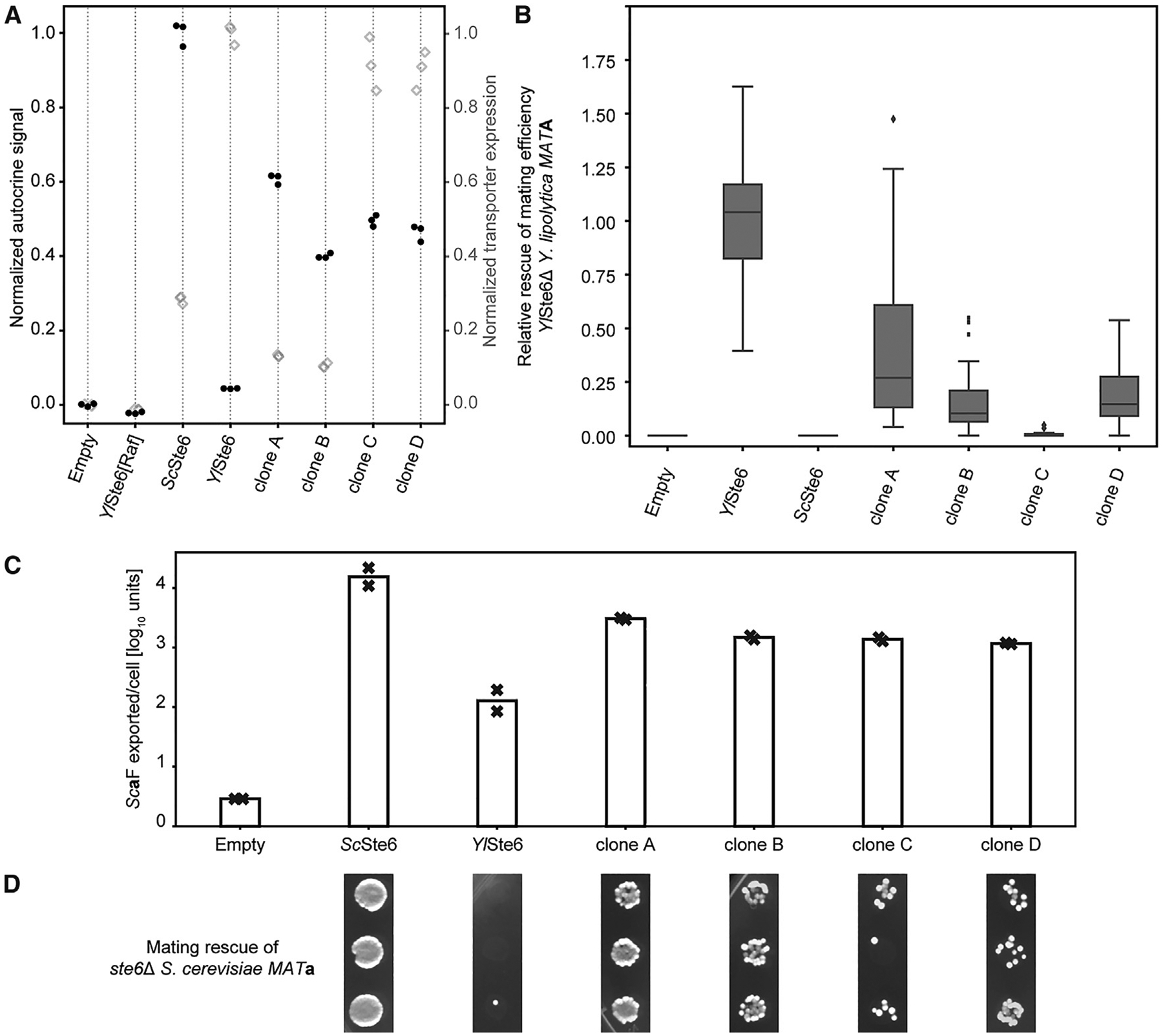
Autocrine Selection Produces Transporters with Increased S. cerevisiae a-Factor Transport (A) Four clones (A and B from the TM4–6 library and C and D from the TM10–12 library) were re-introduced into naive autocrine strains (that have not experienced FACS) to verify that increased autocrine signal is conferred by mutations in the clones. Each sample was measured by flow cytometry in biological triplicate populations (n > 25,000), and the normalized medians of the autocrine signal (black solid dots) and transporter expression (gray open diamonds) are plotted. The data were collected in high-throughput autocrine experiments (see STAR Methods) to make sure samples can be consistently compared. Common samples are duplicated in Figure S6A (right). (B) Clones A, B, C, and D have reduced mating efficiency in *Y. lipolytica MAT***A** cells (equivalent to *MAT*a mating type of *S. cerevisiae*) when substituting for *Yl*Ste6, suggesting a reduced ability to transport *Y. lipolytica* a-factor. The efficiency is the number of diploids for a sample relative to the mean number of diploids formed with WT *Yl*Ste6. The boxplots contain data from four different experiments, each with two biological replicates, with the box representing the middle 50%, median marked as a line, and the whiskers representing the outer quartiles of the distribution with individual outliers plotted as filled black diamonds. (C) *Sc*a-factor exported by populations expressing the indicated transporters was measured by an endpoint dilution biological assay in duplicate (crosses, average denoted by bars; units as in Figure 1C). Selected clones A, B, C, and D have increased a-factor export relative to WT *Yl*Ste6. (D) Mating rescue of *ste6*D *S. cerevisiae* strains expressing transporter samples in biological triplicate plated on diploid selective media. Clones give increased rescue of *S. cerevisiae* mating compared with that of WT *Yl*Ste6. See also Figure S4.

We performed a more detailed analysis of the effect of mutations in selected clones. We selected four clones with high autocrine signal (two each from the TM4–6 and TM10–12 libraries) (Figure 4A) to better characterize the increased autocrine reporter signal. To confirm that selection for increased autocrine signal corresponds to transporters with altered substrate selectivity and increased transport of *Sc*a-factor, we used two additional assays: quantifying mating efficiency and the exported a-factor. Cells expressing each of the four selected clones show a roughly 10-fold increase in a-factor export relative to cells expressing unmutated *Yl*Ste6 (Figure 4C), and the expression of these mutants improves mating efficiency relative to cells expressing the WT *Yl*Ste6 (Figure 4D), although the correlation between autocrine signaling and mating efficiency is modest due to the extra steps required for mating. These results demonstrate that the selected clones confer increased a-factor transport.

Mutants that increase the export of *Sc*a-factor could act by at least three mechanisms: increasing the transport rate without altering substrate specificity; reducing transporter specificity to allow efficient transport of both *Sc*a-factor and *Yl*a-factor; or altering specificity to transport *Sc*a-factor better and *Yl*a-factor worse. To distinguish these possibilities, we assayed the *Yl*a-factor transport function of the mutant transporters in *Y. lipolytica*. Like *S. cerevisiae ste6*D mutants,*Y. lipolytica ste6*D *MAT*A (equivalent to the *MAT*a mating type of *S. cerevisiae*) strains mate very poorly. We can restore the mating phenotype by expressing *Yl*Ste6 on a replicating plasmid and therefore test the pheromone export activity of a mutated transporter expressed from a corresponding plasmid (Figure S4). Three of the four selected clones expressed in *Y. lipolytica ste6Δ* strongly reduced mating efficiency compared with expression of unmutated *Yl*Ste6 (Figure 4B), suggesting that these mutants have reversed the substrate specificity of *Yl*Ste6 rather than reducing substrate selectivity to allow efficient export of both pheromones.

### Individual Mutations from Selected Clones Increase Pheromone Export

**Figure 5.**
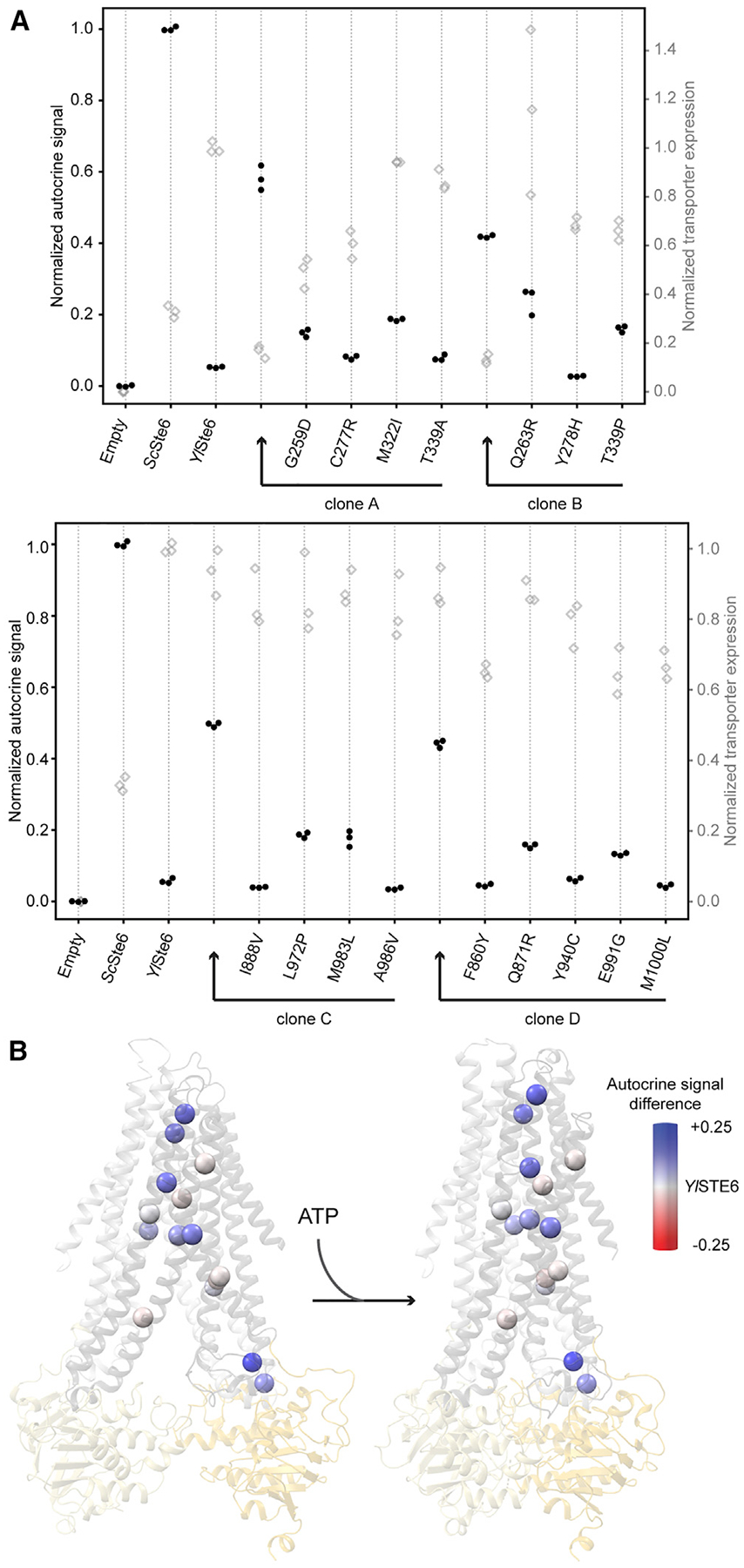
Effects of Individual Mutations from Selected Clones are Neutral to Positive (A) Mutations in clones A, B, C, and D were tested as individual mutations by the flow-cytometry-based autocrine assay, showing that many single mutations increased transport activity. Each sample was measured in biological triplicate populations (n > 25,000), and the normalized medians of the autocrine signal (black solid dots) and transporter expression (gray open diamonds) are plotted. Clones A and B from TM4–6 libraries (top) and clones C and D from TM10–12 libraries (bottom) are plotted alongside their corresponding single mutations for ease of comparison. The data were collected in high-throughput autocrine experiments (see STAR Methods) to make sure samples can be consistently compared. Common samples from the top graph are duplicated in Figure 6A (top) and Figure S6A (left); and from the bottom graph are duplicated in Figure 6A (bottom). (B) All mutations contained in clones A, B, C, and D are plotted as C*α* spheres on *Yl*Ste6 homology models and colored by autocrine signal difference values (subtracting autocrine signal of WT *Yl*Ste6). Table S1 records the values used for the structural models. See also Figure S5 and Table S1.

We examined the effect of individual amino acid substitutions on *Yl*Ste6 activity and the interaction between these mutations. The four clones tested above (clones A and B from TM4–6 and clones C and D from TM10–12) each contain between 3 and 5 mutations, and we next tested the effect of each mutation in isolation. We introduced the individual mutations in *Yl*Ste6 by using directed mutagenesis and measured the autocrine signal by flow cytometry with the same conditions used for selection (Figure 5A). We subtracted the WT *Yl*Ste6 signal from the autocrine signal conferred by *Yl*Ste6 transporters with single mutations and considered this difference in signal to be the contribution of each mutation to a-factor export (Table S1). Each clone has at least one mutation that increased autocrine signal when present in isolation. No singly mutated transporter is much worse than WT *Yl*Ste6: half the mutations—C277R, Y278H, I888V, A986V, F860Y, Y940C, and M1000L—were nearly neutral or mildly deleterious.

Plotting the normalized autocrine signal of these single mutations on structural models reveals that most mutations that increase the autocrine signal localize to the TMD cavity (Figure 5). This is true across all four clones, with positions of the TMD cavity buried in the lipid bilayer containing six mutations that increase the autocrine signal: M322I, T339P, Q871R, L972P, M983L, and E991G. We note that four of these six mutations cause substantial changes in the sidechain. However, two strongly beneficial single mutations, G259D and Q263R, are near the coupling helices that are the structural contacts between the TMDs and NBDs. The coupling helices are important in the allosteric communication of substrate binding to the NBDs that underlies the substrate-stimulated ATPase activity that is conserved across ABC transporters [31, 32].

### Mutations Have Additive Effects on Transport Activity

We analyzed the interaction between individual mutations in a given clone to understand whether the autocrine signal of a clone depends on the specific combination of mutations. The effect of individual mutations can be positive, neutral, or negative, with the last two classes potentially hitchhiking with beneficial mutations. Doubles or triples of mutations could produce larger or smaller increases in the autocrine signal than the sum of their individual effects. In extreme cases, adding individually neutral or deleterious mutations could enhance the signal produced by other mutations. None of the mutations in the four tested clones, A, B, C, and D, are strongly deleterious, and therefore, we are restricted to testing the effect of nearly neutral mutations in combination with other neutral or beneficial mutations.

**Figure 6.**
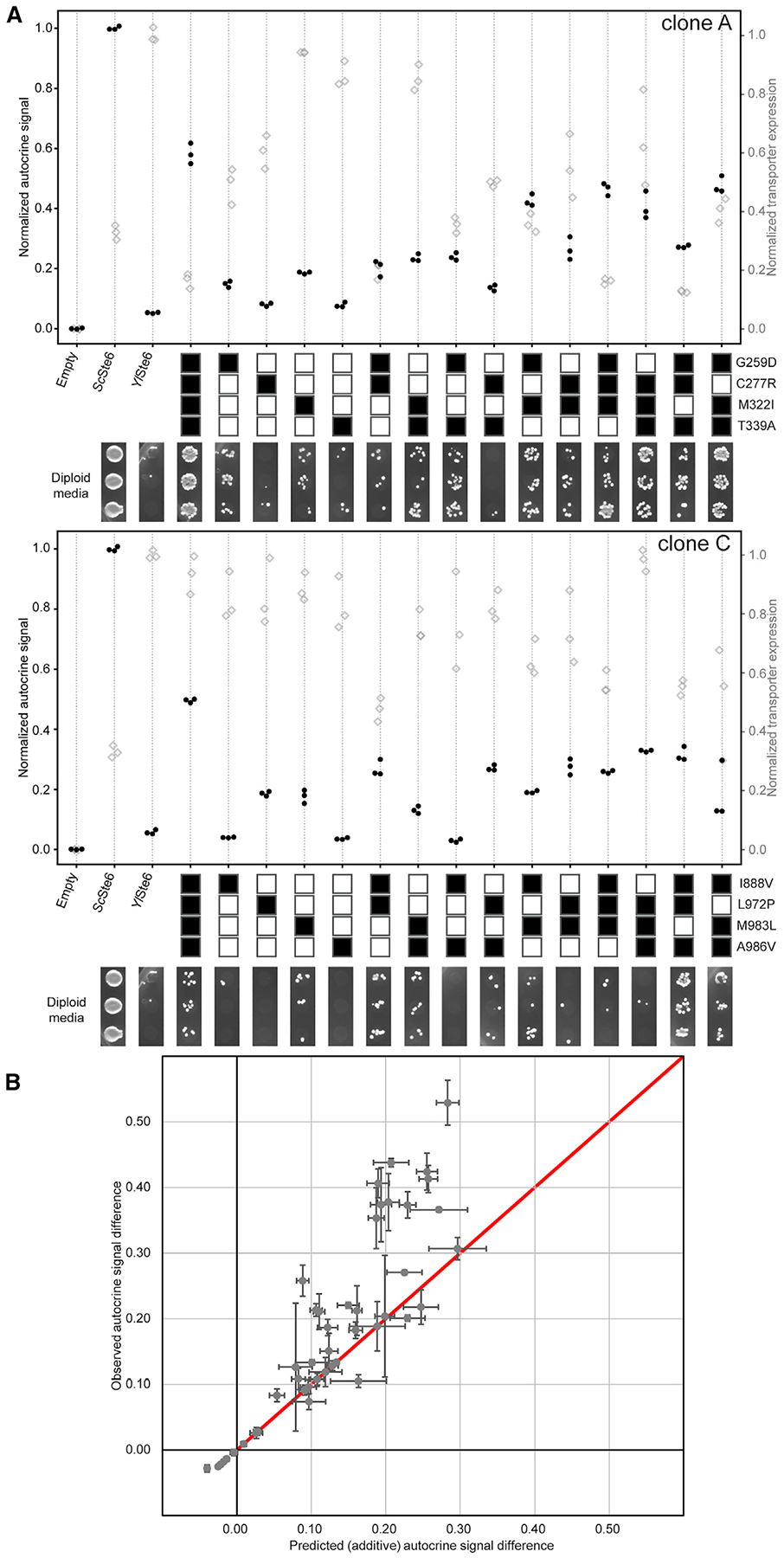
Mutations in Selected Clones Have Roughly Additive Effects on the Autocrine Signal (A) Normalized autocrine signal (black solid dots) and transporter expression (gray open diamonds) of all possible combinations of the mutations found in clones A and C were measured by using the flow-cytometry-based autocrine assay with biological triplicate populations (n > 25,000) for each sample. Combinations of mutations from clone A (top) and clone C (bottom) are represented by a series of boxes; filled boxes represent the presence of a mutation. The data were collected in high-throughput autocrine experiments (see STAR Methods) to make sure samples can be consistently compared. Common samples from the top graph are duplicated in Figure 5A (top) and Figure S6A (left); and from the bottom graph are duplicated in Figure 5A (bottom). Mating activity for cells expressing *Yl*Ste6 containing each combination of mutations is displayed below the autocrine data. The mating data for both panels were collected in a single experiment (see STAR Methods), and thus the same *Sc*Ste6 and *Yl*Ste6 expressing controls are shown in both panels for ease of comparison. (B) Data compared to an additive model for the autocrine signal difference (subtracting autocrine signal of WT *Yl*Ste6). The model, shown as a red line, is such that the autocrine signal difference from a multiple mutant is the sum of autocrine signal difference of all single mutations it contains. The data are shown with error bars representing standard deviation of autocrine signal difference medians of biological triplicate populations. See also Figure S6 and Mendeley Source Data File 4.

To understand the interactions among the mutations in clones that gave a high autocrine signal, we built all possible combinations of mutations contained in clones A and C, from the TM4–6 and TM10–12 libraries, respectively, which each contained four mutations, and a subset of the combinations in clones B and D, which contained three and five mutations, respectively. We tested the combinations by using the autocrine signal detected by flow cytometry (Figures 6A and S6A). If mutations interact additively and the autocrine signal is linearly proportional to Ste6 transport activity, the signal of multiple mutations should equal to the sum of signals of each of the mutations in isolation. Figure 6B plots the signal from all combinations of mutations we tested with the observed value on the y axis and the sum of single mutation contributions on the x axis. Given the error in our measurements and our uncertainty in the relationship between autocrine signaling and transporter activity, our data are consistent with beneficial mutations being roughly additive in their contribution to the autocrine signal. We cannot exclude the possibility that the autocrine signal as a measure of transport activity might be a non-linear transform of an underlying additive property [33]; however, we choose to make the conservative assumption that signaling is proportional to transporter activity [34]. We note that we see little dependence of autocrine signal on transporter expression (Figure S2F) and thus assume there are saturating levels of transporter on the plasma membrane. The mutations in selected clones increase the autocrine signal in an additive manner, explaining the lack of strongly deleterious mutations that would adversely affect the autocrine signal. The mating efficiency of the constructs that contain combinations of mutations from clone A or C are consistent with the measured autocrine signal (Figure 6A) and support the inference that combining beneficial mutations increases a-factor transport activity. The additive nature of mutation effects highlights the fact that most single-step evolutionary paths for clones A and C are either neutral or adaptive.

When mutations are in the same clone, our enrichment for autocrine signaling selects for mutations that interact at least additively with each other. We investigated the interaction of mutations that were selected independent of each other. The clearest example is the interaction of mutations that were independently selected and lie in different parts of the protein. We therefore measured the autocrine signal of the four possible cross-library chimeras of TM4–6+TM10–12 clones: A+C; A+D; B+C; and B+D. The resulting transporters contain combinations of mutations that are beneficial but were not all selected together. As measured by flow cytometry, all the chimeras had significantly lower autocrine signal than the additive expectation (Figure S6B). To further test mutation compatibility, we made chimeras of single beneficial mutations from different clones and compared them to mutations from the same clone. Some chimeras of mutations across clones (A:G259D + C:L972P) are incompatible with each other and produce an autocrine signal much lower than the additive expectation, whereas other pairs (B:Q263R + D:Q871R) are as compatible with each other as mutations from the same clone (Figure S6B). Thus, not all combinations of strongly contributing mutations are additive, and by selecting on the combined effect of multiple mutations, our selection was biased toward clones that have particular combinations of mutations with high autocrine activity.

Although we used only one round of mutagenesis to produce and then select clones that carried multiple mutations, we can still infer possible evolutionary trajectories. Having tested all possible combinations of the component mutations in clones A and C (Figure 6A), we can evaluate the autocrine signal at every step of all 24 possible evolutionary trajectories that lead from the wild-type *Yl*Ste6 to each of clone A and C (Figure S6C, left and right, respectively). Accounting for the uncertainty of our measurements of the autocrine signal (~ 0.02), all trajectories (except 2 for clone C) increase the autocrine signal with each step, making them paths that avoid less-fit evolutionary intermediates. The availability of so many continuously upward trajectories reinforces the idea that it is easy to evolve the substrate selectivity for ABC transporters.

## DISCUSSION

ABC transporters are found in all extant organisms and transport a very wide range of substrates, suggesting that their substrate specificity evolves readily. We investigated the substrate specificity of a member of the ABC transporter family that exports mating pheromones from the cytoplasm of fungal cells. *Y. lipolytica* last shared a common ancestor with *S. cerevisiae* 320 mya and has an a-factor that differs in length, sequence, and amino acid composition from *S. cerevisiae*’s a-factor. The sequence identity between the *Y. lipolytica* and *S. cerevisiae* Ste6 proteins (31%) is only slightly greater than that between these two proteins and ABC transporters that export small molecules (23% and 25% to human P-glycoprotein) or antigenic polypeptides (18% and 17% to human TAP). Using an assay that enriches for cells with increased pheromone transport, we selected mutants of the *Yl*Ste6 protein that efficiently transport the *Sc*a-factor. These mutants contain multiple mutations that independently and approximately additively contribute to increased pheromone transport. The multiple mutants that improve pheromone transport in *S. cerevisiae* impair transport in *Y. lipolytica*, implying that they alter rather than relax the transporter’s substrate specificity and thus reverse a substrate specificity that has evolved over approximately 320 Ma [27].

To understand the evolution of protein function and specificity, we must find the amino acid substitutions that alter these properties and investigate the interactions between individual mutations. But it is difficult to distinguish the mutations that alter function from background variation that occurs in the course of evolution, and this challenge grows larger as the evolutionary distance between the proteins increases. High-throughput functional screening of comprehensive, single-substitution libraries of mutant proteins elucidates the role of amino acids at individual positions in a protein [35], and this approach can be extended to comprehensively analyze the role of combinatorial variation in a small number (%5) of interacting positions [36]. These exhaustive screens reveal the local response of function to mutation. In contrast, random, combinatorial mutagenesis selects for novel or altered functions that might depend on multiple mutations over a wide range of positions, and thus, such selections cover only a small fraction of a high-dimensional sequence space. We took advantage of the well-studied molecular mechanisms of the yeast pheromone response to design a high-throughput, flow-cytometry-based selection for a pheromone exporter’s function by coupling its activity to the expression of a fluorescent reporter. Our approach could be easily modified to test the sequence-function relationship of other proteins in the mating pathway, including the specificity of the pheromone receptors [37].

Type I ABC exporters are a large subfamily that share structural and sequence similarity although the specific sequence features responsible for substrate selectivity remains elusive. Type I exporters are involved in the drug resistance of pathogens [7, 38] and cancers [6], and mutations in several human homologs cause inherited diseases, including cystic fibrosis [39–42]. This diversity in physiological roles highlights the variation in the substrates that different paralogs transport and suggests that understanding substrate selectivity could lead to the production of more potent and specific inhibitors of ABC transporters. Three previous types of study have given information about the positions that contribute to substrate binding of type I exporters: substrate-bound structures [4, 31, 43]; crosslinking of modified substrates to the exporters [10, 44, 45]; and natural allelic variants that affect the peptides transported by TAP [8, 44, 46]. Collectively, these studies identify residues located throughout the TMD cavity with a higher density near the cavity’s apex. Although informative, none of this work took a systematic approach toward identifying residues whose mutation alters substrate specificity.

To remedy this deficit, we developed a FACS-based selection on autocrine signaling that could identify combinations of mutations that increased the transport of *Sc*a-factor by *Yl*Ste6. The fraction of our *Y. lipolytica* Ste6 libraries that gives increased export of *S. cerevisiae* a-factor allows us to make a rough estimate of how many positions in Ste6 can mutate to alter substrate specificity. In our experiments, we mutagenized a contiguous region of about 160 amino acids and obtained clones that contained between three and five mutations (Figure S3C) that contributed roughly additively to improved *Sc*a-factor export by *Yl*Ste6. To estimate the target size for mutations that produced the improved *Sc*a-factor export we observed, we make three assumptions: that it takes mutations at three positions to improve pheromone export; that each position requires a specific mutation to improve export; and that all mutations at the third position of a codon are synonymous. In this model, there are 669,920 combinations of three out of 160 positions where mutations can occur (160-choose-3), and for each set of three positions, there are six nucleotide positions (two per codon) with three alternate bases, leading to 6^3^ = 216 different combinations of non-synonymous single-base substitutions for each group of three positions, giving a total of 1.45 × 10^8^ different triple mutant clones. In a library of roughly 10^5^ mutant clones, we found about 50 independent selected clones with strongly improved autocrine signaling, suggesting that 0.05% of the mutant clones improve pheromone export. This implies that roughly 0.0005 × 1.45 × 10^8^ = 72,500 triple mutants would satisfy our selection. If only one mutant amino acid at each codon can lead to improved pheromone export, this value implies that 77 out of the 160 positions can produce such mutations (77-choose-3 = 73,150). Even if two possible mutant amino acids at each position would improve *Sc*a-factor export (leading to 2^3^ = 8 different combinations of favorable mutations at each set of three positions), the inferred number of relevant positions is only reduced to 39. Therefore, for each TMD, we expect about 50 positions to influence substrate selectivity, leading to about 100 positions in a full transporter. Even if these calculations err by an order of magnitude, our estimate suggests that there are an enormous number of mutational trajectories that could alter the specificity of ABC transporters. The fraction of clones that show strong expression was lower in libraries produced by a higher level of mutagenesis, suggesting a balance between accumulating mutations whose effects are roughly additive in providing a selective benefit without including a strongly deleterious mutation (including substitutions that prevent protein folding or function and frameshift and nonsense mutations). The cost of mutagenesis was confirmed by measuring transporter expression in unselected libraries that were mutagenized to various extents (Figure S2E). As expected, median transporter expression falls as the average mutation count of the library increases.

**Figure 7.**
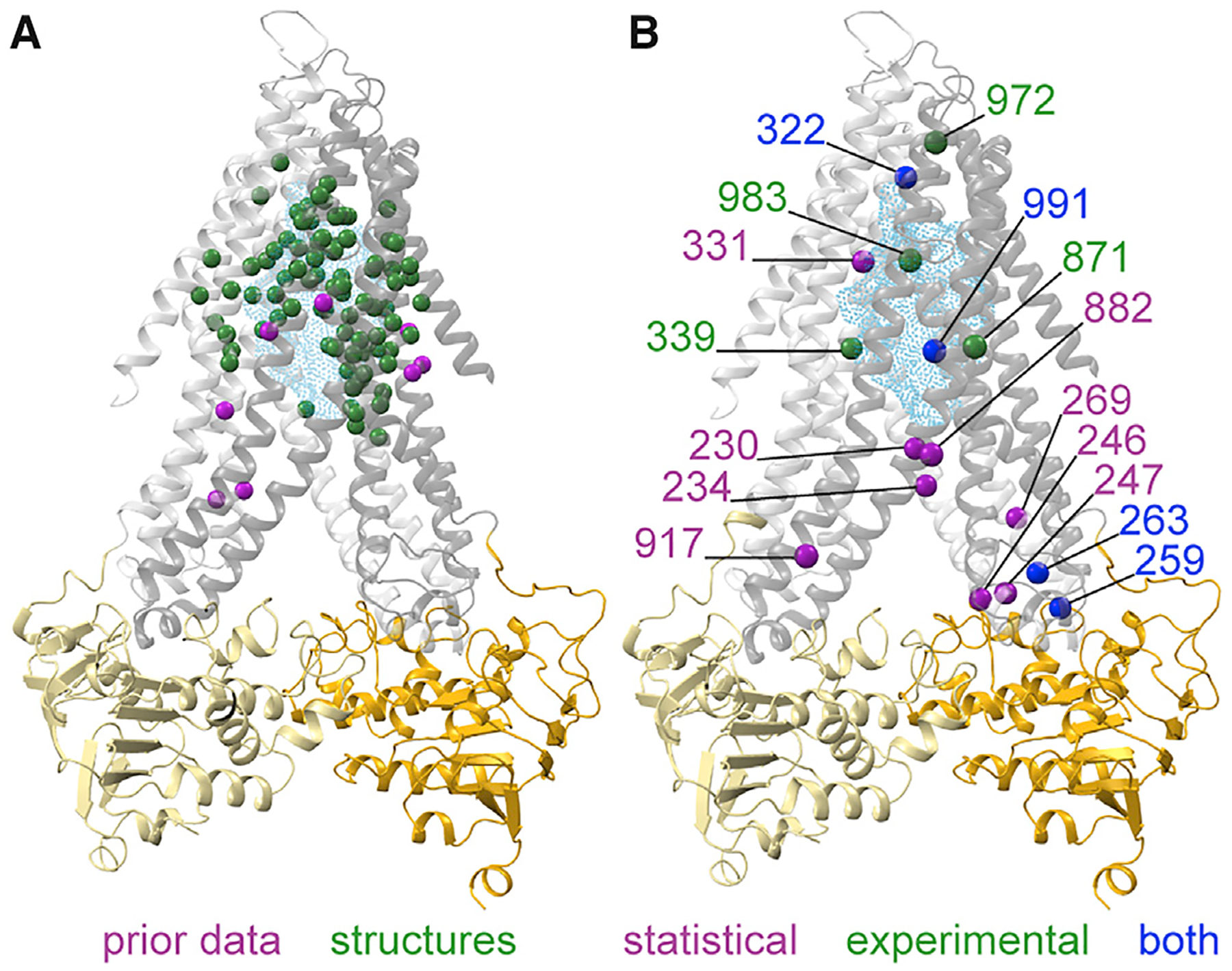
Statistically Enriched and Experimentally Validated Positions from Clones with Increased Autocrine Signal Compared to WT YlSte6 (A) Inward-open homology model of *Yl*Ste6 reproduced here from Figure 2C for comparison. Green spheres highlight positions proximal to substrate density, and magenta spheres highlight positions homologous to residues in TAP and P-gp shown to affect substrate recognition by crosslinking, allelic variants, and constructed mutations. The cyan cloud represents the cumulative density of the substrates listed in the legend to Figure 2C. (B) Identified positions that contribute to increased autocrine signal from mutant libraries of *Yl*Ste6 TM4–6 and TM10–12 are mapped onto the inward-open homology model of YlSte6; positions were identified by statistical enrichment (magenta), experimental validation (green), or both (blue). These positions are distributed across the TMD with the cyan cloud in the TMD lumen representing cumulative substrate density from aligned substrate-bound structures of P-gp, MsbA, and MRP1 as in Figure 2C. See also Figure S7.

We used two forms of analysis to identify positions where mutations contributed to increased transport. Our statistical analysis identified 12 positions that were mutated much more often than expected by chance. In addition, our experiments on individual mutations identified 8 positions where mutations increased *Sc*a-factor export. All four of the statistically enriched positions that overlap with the experimentally tested set were found to increase autocrine signal, meaning that we found 16 positions (10 in TMs 4–6 and 6 in TMs 10–12) where mutations were either demonstrated or inferred to increase *Sc*a-factor export (Figure 7). The simplest hypothesis for the increased substrate selectivity in the selected clones would be that critical residues in *Yl*Ste6 must mutate to the corresponding amino acid in *Sc*Ste6. We binned the mutations at our 16 identified positions into 3 classes—mutations of a residue from *Yl* to *Sc* (1 position), mutations from a residue that was identical in *Yl* and *Sc* (2 positions), and mutations from *Yl* to a residue different from the one in *Sc* (13 positions) (Figure S5). Thus, we found only one position where some of the mutations changed the amino acid in *Yl*Ste6 to the one in *Sc*Ste6, and most mutations are to amino acids that are not present in either *Yl*Ste6 or *Sc*Ste6. Furthermore, 10 of the 16 identified positions have more than one new residue at the same position (Figure S7C), suggesting that multiple substitutions at the same site can affect substrate selectivity.

We asked how many of the 92 unique selected clones contained a mutation in these 16 identified positions (Figure S7A). All but 5 of the 59 clones from the TM4–6 libraries contain a mutation in at least 1 of the 10 identified positions within TM4–6. In contrast, about one-third of the TM10–12 clones (12 of 33) do not contain any of the 6 identified positions within TM10–12. We believe that this reflects the weaker statistical inference that is possible from the smaller number of clones that we isolated from this library. For example, only 1 of the 4 experimentally validated positions are also statistically enriched, compared with 3 of 4 positions in the TM4–6 clones (Figures 7 and S7B). Using a single round of mutagenesis followed by cycles of enrichment favors mutations that have beneficial effects as single mutations and are compatible with a variety of other mutations, both neutral and beneficial. The limited size of our randomly mutagenized libraries might have prevented the identification of mutations that are individually weak(er) or contribute in more limited backgrounds. The modest number of unique clones that we identified means that we do not believe we have found all positions where mutations improve *Sc*a-factor export. That 10 of the 16 identified positions show mutations to more than one amino acid suggests that there are many positions where at least two amino acid substitutions are beneficial. These two factors and the assumptions in our calculation lead us to argue that there are at least 16 and as many as 100 positions in *Yl*Ste6, where mutations can lead to a substantial increase in the ability to export *Sc*a-factor.

We can roughly categorize the positions where mutations improved pheromone export into two groups: one at the TMD cavity buried in the membrane and the other near the coupling helices that connect to the NBD (Figure 7). Substrate-bound structures of homologous type I exporters with diverse substrates and competitive inhibitors [4, 31, 43, 47, 48] identify interacting residues that position substrate at the apex of the inward-open cavity (Figure 2C). Besides direct interactions that stabilize substrate binding, TM4 and TM10 undergo conformational changes in response to cognate substrate binding [31]. We speculate that mutations in the *Yl*Ste6 TMD cavity contribute to substrate binding, either by direct sidechain interactions or by contributing to the flexibility of TMs needed for conformational changes (Figure 7). Substrate binding enhances ATP-dependent NBD dimerization, observed as substrate stimulation of ATPase activity, and is allosterically communicated by the coupling helices that form tertiary contacts between the TMD and NBD [31, 32]. Our second group of positions, near the coupling helix, may affect the allosteric pathway between the TMD and NBD to increase a-factor transport. ABC exporters contain two pairs of coupling helices that connect the two TMDs to the two NBDs. Mutations identified a role for coupling helix 1 of TAP1 (between TM2 and TM3) (Figure 2D) in sensing and transport of antigenic peptides [49]. Although coupling helix 1 is not contained in the mutagenized regions of our libraries, simulations of P-gp [32] suggest an influence of coupling helix 2 (located between TM4 and TM5), which was mutated in our clones, on substrate binding and NBD dimerization. Incorporating the statistically enriched positions, both the TM cavity and coupling helix groups gain more members (Figure S7B). Our data suggest that mutations over much of the TMDs affect transport activity in two ways: by directly affecting substrate binding and by affecting the coupling between substrate binding and ATP hydrolysis. More generally, in ABC exporters, we speculate that the target size for the evolution of substrate selectivity covers a large part of the TMD and might involve both substrate binding and allostery.

Although *Yl*a-factor and *Sc*a-factor differ in length and amino acid composition and have no recognizable sequence homology, we were able to identify mutations that greatly improved *Yl*Ste6’s ability to transport *Sc*a-factor. These mutations lie on the surface of the TMD cavity, where variation across the ABC family allows its members to recognize substrates that differ greatly in their size, structure, and chemical properties. Recent cryoelectron microscopy (cryo-EM) studies of different ABC exporters [43, 50, 51] have reached consensus on the major conformational states of the substrate export cycle. The transition between inward-open and outward-open states is expected to occur via two successive transitions: (1) substrate binding induces a conformational change that leads to dimerization of the NBD and (2) NBD dimerization induces opening of the substrate-binding cavity to the external face of the plasma membrane. In studies on homologous transporters, mutations in the TMD cavity can either directly contact the substrate [44], alter the flexibility of TM helices [31], affect the contacts between TM helices [52], or potentially contribute to substrate selectivity allosterically by affecting the coupling helices and NBD interactions [32, 53].

We argue that the large target size and additive interaction of mutations make the evolution of substrate specificity in ABC transporters different from that of most enzymes. In contrast, work on several enzymes suggests that strong epistasis prohibits most evolutionary trajectories that would allow them to act on new substrates. As an example, analysis of b-lactamase’s ability to hydrolyze novel β-lactam antibiotics finds that most trajectories are prohibited because they pass through intermediates that reduce the enzymatic activity toward the novel substrate [54]. The dependence of a mutation’s effect on both the remainder of the protein sequence and the order of mutations further highlights the importance of both positive and negative epistasis in restricting the available trajectories for novel functions to evolve in enzymes [55, 56]. Studies of histidine kinase-response regulators and antigen-antibody protein interfaces also reveal strong epistatic contributions to mutations that maintain or alter binding interfaces [36, 57]. In contrast, a large number of positions can mutate to produce additive effects on the substrate specificity of ABC transporters, and we propose that these relaxed molecular constraints underlie the enormous expansion of this family of primary transporters and allow them to be maintained in every branch of life.

## STAR★METHODS

### LEAD CONTACT AND MATERIALS AVAILABILITY

Further information and request for resources and reagents should be directed to and will be fulfilled by the Lead Contact, Andrew Murray (awm@mcb.harvard.edu). All unique/stable reagents (strains and plasmids) generated in this study are available from the Lead Contact without restriction.

### EXPERIMENTAL MODEL AND SUBJECT DETAILS

#### S. cerevisiae

The budding yeast *Saccharomyces cerevisiae* strains used in this study are derived from the standard w303 strain (listed in Table S2). Strains were generally grown in YPD or CSM media at 30°C or as specified in the Methods Details. Established protocols to work with these species were adapted as described in the Method Details section below.

#### Y. lipolytica

The alkane-using yeast *Yarrowia lipolytica* strains used in this study are derived from the standard CLIB122 strain (listed in Table S2). Strains were generally grown in YPD or CSM media at 30°C or as specified in the Methods Details. Established protocols to work with these species were adapted as described in the Method Details section below.

### METHOD DETAILS

#### Strains and plasmids

All yeast strains were derived from either a *MAT*a W303 haploid (*MAT*a; *ade2–1*; *can1–100*; *leu2–3,112*; *his3–11,15*; *ura3–1*; *trp1–1; bud4-W303*) or a *MATα* W303 haploid cell (*MATα*; *BUD4*; *can1–100*; *leu2–3,112*; *his3–11,15*; *ura3Δ*) (Table S2). Strains were transformed using the LiAc-mediated chemical transformation protocol [66]. Selective markers are derived from wild-type versions of the *S. cerevisiae* genes, with 300–500 bp of homology flanking the marker for targeting genomic integration by homologous recombination. The *Y. lipolytica* pair of mating strains (ML16507 and ML16510) were a gracious gift from Joshua Truehart (DSM Ltd) and are derivatives of the sequenced CLIB122 strain [67]. Genomic transformation of *Y. lipolytica* was done using reported protocols [68].

The *S. cerevisiae* plasmid used to test the activity of heterologous pheromone transporters, pSS006, was used to construct strains in *Sc*aF-collection and mating experiments. Plasmid pSS006 was constructed by introducing the following elements into pUC19 (ATCC 37254) between the BstBI and AatII restriction sites: the upstream flanking region of *ScSTE6* locus (for homology), the *S. cerevisiae GAL1* promoter (*P*_GAL1_), NdeI and NotI restriction sites for ORF cloning, an in-frame thrombin cleavage site followed by the coding sequence of EGFP (to quantify expression), the *S. cerevisiae STE6* terminator (*T*_STE6_), *P*_TEF1_*-KanMX-T*_TEF1_ (providing resistance to G418 (Thermo Fisher, Ref 11811031)), the downstream flanking region of *ScSTE6* locus (for homology), and *CEN6/ARSH6* (from the pRS41*x* plasmid series [69]). The *S. cerevisiae* plasmid developed for transporter expression in the autocrine experiment, pSS021, was also used for *Sc*aF-collection and mating experiments with clones from the selection. Plasmid pSS021 was derived from pRS413 (*HIS3*, *CEN6/ARSH4*; ATCC 87518 [70];), with the expression locus inserted between the XhoI and SacI restriction sites. The inserted cassette includes the *S. cerevisiae GAL1* promoter (*P*_GAL1_), NdeI and NotI restriction sites for open-reading frame (ORF) cloning, an in-frame thrombin cleavage site followed by the coding sequence of ymKate2 tag [71] to quantify expression, and the *S. cerevisiae ADH1* terminator (*T*_ADH1_). The Ste6 genes from selected species were PCR amplified from genomic DNA and Sanger sequenced to confirm no differences relative to the reference sequence available at NCBI. Mutations T613A and S623A were made in *Sc*Ste6 to improve the lifetime of the protein. They are reported to reduce the transporter recycling mediated by phosphorylation-induced ubiquitination [72]. Ste6 homologs (Table S3) were inserted into pSS006 and pSS021 by Gibson assembly by digesting the backbone with NdeI and NotI. *Y. lipolytica* plasmid PMB8369 was a gracious gift from Joshua Truehart (DSM ltd.), and C-terminally ymKate2-tagged transporter ORFs (as made in pSS021 above) were introduced into PMB8369 at the NotI site by Gibson assembly. All plasmid ORFs were confirmed by Sanger sequencing.

#### Collection and bioassay for a-factor

a-factor was isolated from cultures of *S. cerevisiae* by taking advantage of its hydrophobicity as previously described [73]. Media used in this work are modified from Yeast extract, Peptone, (YP) or Complete Synthetic Medium (CSM) [74]. Briefly, overnight cultures of *MAT*a cells (expressing different transporter homologs) grown at 30°C in YP with 3% (v/v) glycerol or CSM-His with 3% (v/v) glycerol plus 0.05% (w/v) dextrose were harvested and cells were inoculated at a density of 10^8^ cells in 5 mL of collection medium (CSM with 2% (w/v) D-galactose (D-Gal), 1% (w/v) D-raffinose (D-Raf), and 0.75 μM *Sc*a-factor (peptide WHWLQLKPGQPMY, ordered from Bio-Synthesis, https://www.biosyn.com). Galactose induces Ste6 expression and *α*-factor induces maximal a-factor expression. In *S. cerevisiae*, a downstream response of the pheromone signaling cascade is a Far1-dependent cell-cycle arrest. The cell-cycle arrest is essential to the cellular transcriptional response that drives increased pheromone output, cell polarization, and subsequent mating by cell-cell fusion. The a-factor collection cultures were incubated in a roller drum at 30°C in a 14 mL (17×100 mm) polystyrene culture tube with the tubes slanted to maximize surface area of the tube exposed to culture. The polystyrene surface acts as an affinity resin for the hydrophobic a-factor secreted from cells. After 7–8 h, an aliquot of the culture was used to measure cell density using a Coulter Counter (for normalization of collected a-factor to the cell density of the cultures) and GFP fluorescence (488nm laser excitation, baseline subtracted with the signal from a no-transporter culture) on a High Throughput Sampler-enabled (HTS) Fortessa. The rest of the culture was discarded, and the culture tubes were washed twice by adding 5 mL sterile water, vortexing and aspirating. The empty culture tubes were spun at 1,000 g for 2 min and remaining water was aspirated. The culture tubes (caps left ON loosely) were left to dry at room temperature. One mL methanol was added to each tube and the caps were sealed tight. The tubes were briefly vortexed and left at 4°C overnight to elute the a-factor extract from the walls of the tube. The extract was transferred to labeled microfuge tubes and vacuum evaporated to dryness and then resuspended in 40 μL methanol (a 125-fold concentration from 5 mL cultures). Two-fold serial dilutions, to a maximum dilution of 33,000-fold, were prepared from all samples in methanol and 5 μL of each dilution spotted on YPD plates (8 spots per plate, separated to avoid interference by diffusion). An overnight culture of *MATα*-cells (ySS209) at 10^6^-10^7^ cells/mL was sprayed onto the spotted plates using an atomizer (Oenophilia, REF 900432) to generate a uniform lawn. After the plates were dried, they were incubated at 30°C overnight, and imaged. The end-point dilution of a-factor in the extract was the lowest extract concentration that still prevented growth of the *MATα*-cell lawn on the spot. The a-factor exported per cell was determined as the last fold-dilution of extract that still arrests cell growth divided by the number of cells in the collection culture at the end point (7–8 h). The data are reported in log_10_ units because the precision of measurements is restricted by serial dilution.

#### S. cerevisiae 96-well plate mating assay

The *S. cerevisiae* mating assay was modified from a classic quantitative filter mating assay [75] to produce a high-throughput, semiquantitative test. *MAT*a-cells (ySS405) containing pSS021 (*HIS3/CEN*) or pSS006 (*KanMX/CEN*) derivatives containing the transporters to be tested were pre-cultured overnight at 30°C in selective medium (2% (v/v) glycerol + 0.05% (w/v) dextrose) in a 96-deep-well block with biological triplicate colonies, while a single large culture of *MATα* cells (ySS319) was started in YPD to use as a mating partner. The cells were pelleted, washed in water and 5 × 10^6^ cells of each mating type were mixed in a flat-bottom 96-well plate in 200 μL CSM + 2% (w/v) D-galactose and the plate was incubated at 30°C for 6 h to allow the cells to settle and form zygotes. The D-galactose is sufficient for saturating transporter induction of the *GAL1* promoter (*P*_GAL1_). After mating, 15 μL of 20% (w/v) dextrose stock was added to each well, the plate was sealed with breathable film and agitated for 250–300 minutes to allow zygotes to reenter the cell cycle. The samples were pelleted, medium was discarded and the cells were resuspended in 150 μL sterile water and then printed on media that selected for haploids (CSM-His for pSS021 plasmid containing *MAT*a cells, and CSM-Ade for *MATα*) or diploids (CSM-His-Ade) and incubated at 30°C until there was clear colony growth. For mating experiments in Figure 1C, *MAT*a strains containing pSS006 plasmids were *his*^*-*^ and the experiments were therefore plated on CSM-Lys and CSM-Lys-Ade to select for *MAT*a haploids and diploids. The plates were imaged, and images were processed in Fiji (ImageJ) [63, 64].

#### Error-prone PCR mutagenesis and library construction

The error-prone PCR mutagenesis protocol reported in [76] was modified, using various concentrations of Mn^2+^ as the mutagenic agent. Briefly, the protocol provided for Taq polymerase (NEB, Cat# M0273S) was followed using 0.5 mM, 0.25 mM, 0.125 mM or0.0625 mM MnCl_2_ to mutagenize TM4–6 (residues 199–359; primers oSS10_062 and oSS10_063) or TM10–12 (residues 851–1012, primers oSS10_089 and oSS10_090) (Table S4). The reactions were run over 30 cycles with 10–20 ng of template DNA (WT *YlSTE6* in plasmid pSS021) in a Bio-Rad S1000 thermal cycler. The number of mutations per clone was estimated by Sanger sequencing ~10 clones from each Mn^2+^ concentration. The mutation rate increased monotonically with Mn^2+^ concentration (Figure S2B). AboutȈ90 clones from a TM4–6 library mutagenized with 0.5 mM Mn^2+^ were sequenced to confirm our estimate of mutation rate. This study ran six independent enrichment experiments of libraries, three each for TM4–6 and TM10–12. The three enrichments for each region were from independent PCR mutagenesis; two enrichments with independent libraries mutagenized with 0.5 mM Mn^2+^, and the third enrichment with libraries mutagenized with 0.25 mM and 0.125 mM Mn^2+^.

Our libraries were built *in situ* by co-transforming the mutated region and backbone linear fragments into *S. cerevisiae* and relying on homologous recombination to create circular plasmids containing mutated versions of *YlSTE6* [77]. Primers used for error-prone PCR are homologous to the ends of SnaBI- and EcoRI-digested backbone of *Yl*Ste6[TM4–6]-pSS021 and *Yl*Ste6[TM10–12]-pSS021. The restriction sites were introduced by a modified site-directed mutagenesis protocol [78], where a primer pair is designed to introduce specific mutations on a plasmid by PCR. We used electroporation to transform the linear fragments into *S. cerevisiae* [79], to build as large libraries as possible, with a Bio-Rad pulser set to 2.5 kV, 300 Ω, and 25 μF, which led to an effective 200 Ω sample pulsed for ~4.1 ms and gave 10^4^-10^5^ colonies per mg of transformed DNA. The cells were plated on selective medium, washed off the plate and used as the starting population of our library. Given that our libraries cover a small fraction of the large combinatorial space of mutations in TM4–6 and TM10–12, we decided to build multiple, independent libraries for each region.

#### Building a FACS selection system for a-factor transport

Pheromone export is difficult to select for directly because “success” involves the transport of pheromone from the cytosol to the extracellular medium and a-factor is highly hydrophobic (Figure S1A), adsorbing to glass and plastic. We thus designed an indirect selection, in which a cell responds to the pheromone that it, itself, has exported instead of responding, as cells normally do, to a pheromone from cells of the opposite mating type. This scheme depends on two properties of the pheromone response. First, in*S. cerevisiae* the signaling cascade downstream of the G-protein coupled pheromone receptors—the *α*-factor receptor Ste2 in a-cells and the a-factor receptor Ste3 in *α* -cells—is the same [80], meaning that the a-factor receptor Ste3 can be expressed and functions in an a-cell. Thus, by replacing Ste2 with Ste3 in a-cells, we engineered an a-cell that can detect the pheromone it has exported. Second, pheromone-induced promoters can drive the expression of fluorescent proteins as a reporter for the pheromone-induced signaling cascade [28]. To allow us to turn the pheromone response off after each round of selection, we expressed the a-factor receptor conditionally from the *S. cerevisiae GAL1* promoter (*P*_GAL1_). Transferring cells from galactose- to glucose-containing medium eliminates receptor expression and allows cells to recover from pheromone-stimulated cell cycle arrest. Therefore in ySS491, a *MAT*a strain, the *STE2* locus is replaced by the a-factor receptor, Ste3, expressed under the control of the inducible promoter *P*_GAL1_ [80, 81], and the *LYS2* coding sequence is replaced by ymNeonGreen that is expressed under the control of the pheromone-induced promoter, *P*_FUS1_ [28, 82]. *P*_GAL1_ is converted to an inducible promoter by disabling the positive feedback loop of the galactose regulon by expressing *GAL3* from the *ACT1* promoter [82].

By including bovine serum albumin (BSA, 0.1% w/v), which binds tightly to hydrophobic substances (like the farnesyl group in a-factor) in solution, we prevented a-factor exported from one cell from stimulating neighboring cells that were unable to export a-factor (Figure S2A). Briefly, cells expressing *Sc*Ste6 and *Yl*Ste6 were mixed at [1:1] or [1:10], respectively, and sorted (Fluorescence-Assisted Cell Sorting, FACS) by gating on autocrine signal that was greater than the brightest 1% of *Yl*Ste6 population (Figure S2A). Sorted cells from the mixed populations were identified by unique genetic markers expressed by the *Sc*Ste6 and *Yl*Ste6 strains. Our FACS enrichment protocol leads to a 20-fold enrichment of cells expressing *Sc*Ste6 over those expressing *Yl*Ste6. This test reveals that we can use this system to enrich pooled libraries of mutant transporters (*Yl*Ste6) for those clones that export *Sc*a-factor better by selecting for cells that most strongly express the autocrine reporter.

To select which regions of *Yl*Ste6 to mutagenize, we reviewed mutational analysis [8] and substrate crosslinking [10] on homologous type I ABC exporters like P-gp and TAP, which showed that positions that interact with transport substrates or alter substrate selectivity are present over a large part of the TMD cavity. We also aligned structures of substrate-bound exporters MRP1, P-gp, MsbA [4, 43, 47, 48] to identify residues in the TMD cavity whose side chains lie within 5Å of the transport substrate and thus could also affect substrate selectivity in homologous transporters.

Given the distribution of autocrine signal in the control populations, we expect that a single round of selection is not enough to provide sufficient enrichment of functional transporters. Our system allows for expansion of the selected cells in glucose-containing medium without autocrine stimulation followed by further rounds of FACS-based enrichment after exposure to galactose-containing medium (Figure 2B). The autocrine signal of unselected mutant libraries is generally weaker than that of cells expressing WT *Yl*Ste6 (Figure S2C, lightest blue and dashed green lines respectively). We infer that most mutations are deleterious to transport function, probably by impairing the folding or stability of Ste6, as the transporter expression signal of unselected libraries was lower than populations expressing WT *Yl*Ste6 (Figure S2E). Four rounds of enrichment led to a stronger autocrine reporter signal, corresponding to selecting a subset of the transporter library with increased a-factor export. There was no meaningful change in autocrine reporter signal between the third and fourth rounds of selection for sample libraries (Figure S2C) and we did not want to reduce the diversity of the selected clones, so we used four rounds of enrichment for all selections.

#### Autocrine assay and FACS or flow cytometry

The autocrine strain (ySS491) was constructed to establish an autocrine loop to fluorescently label yeast cells that have a functional pheromone exporter. *Sc*Ste6- or *Yl*Ste6-transformed populations were used as positive or negative controls, respectively, to measure the transporter expression (transporter-ymKate2) and the autocrine signal (ymNeonGreen) with flow cytometry. Figure 2 highlights the separation of the population clouds in a two-dimensional fluorescent color space, with the separation on the autocrine signal (x-axis) being the relevant dynamic range needed for the selections we performed.

Before selecting for autocrine signaling, the cultures were expanded in selective medium (CSM-His to select for the presence of the plasmid), with the smallest bottleneck being around 10^6^ cells, to maintain library complexity. The libraries were expanded in media with 2% (w/v) Dextrose to keep the autocrine system OFF, inoculated at 2 to 5 × 10^5^ cells/mL in 2% (v/v) glycerol plus0.05% (w/v) dextrose to relieve catabolite repression for about 12 hr, and then inoculated in CSM-His plus 1% (w/v) D-galactose, 1% (w/v) D-raffinose, and 0.1% (w/v) BSA at 5 × 10^5^ to 10^6^ cells/mL and shaken at 30°C for 7 h. Cycloheximide (Millipore Sigma C7698) was added to 100 mg/mL to the cultures to “freeze” reporter expression of cells while sorting on an Aria flow cytometer with 488 nm and 561 nm lasers. Control populations, *Sc*Ste6 and *Yl*Ste6, were included in each experiment. Cells were sorted by setting gates for both the transporter expression and the autocrine signal. The sorting gate for transporter expression was set such that only ~5% Autocrine OFF population (*Yl*Ste6 population in D-raffinose medium), a negative control for transporter expression passed, selecting strongly for cells with transporter expression. This was used in combination with a sorting gate for autocrine signal such that ~1% *Yl*Ste6 population, negative control for *Sc*aF transport passed, selecting for increased autocrine signal (Figure 2B). Library populations were sorted to collect enough events to correspond to 1–5 times the estimated library size (10^4^ to 10^5^). Collected cells were inoculated into 2% (w/v) dextrose to expand the population without selection. This enrichment was repeated over four rounds to enrich for transporters that confer higher autocrine signal.

We plated the resulting enriched populations to yield 1500 single colonies and tested these isolated clones individually using the same flow cytometry protocol; more than 95% of these clones showed increased autocrine signaling indicating improved a-factor export (Figures S2D and S3A). To confirm that the increased autocrine signal is due to altered transporter sequences, we isolated plasmids from the selected clones, transformed them into the ancestral version of the autocrine reporter strain. Flow cytometry on these freshly transformed cells revealed that the selected clones encoded versions of the transporter that produced increased autocrine stimulation (Figure S3B). The rank order of the autocrine signal in the original clones correlates well with the signal produced by transforming the isolated plasmids into fresh recipient cells (Figure S3B).

We tested individual clones, by growing them in 96-well plates and measuring their autocrine signal and transporter expression (median of ~30,000 events) by flow cytometry on a High Throughput Sampler-enabled (HTS) Fortessa with 488 nm and 561 nm lasers. Biological triplicates of *Sc*Ste6, *Yl*Ste6 and empty plasmid controls were present in all plates for normalization. Values were scaled between those for *Sc*Ste6 (set to 1) and empty plasmid (set to 0) for autocrine signal; and between *Yl*Ste6 (set to 1) and empty plasmid (set to 0) for transporter expression.

#### Clone isolation and Sanger sequencing

After enrichment, populations were plated on selective plates to isolate clones as single colonies. Clones were expanded in 400 μL CSM-His + 2% (w/v) dextrose medium in 96-deep-well blocks. The samples were pre-cultured at 1 × 10^5^ to 5 × 10^5^ cells/mL in 400 μL 2% (v/v) glycerol plus 0.05% (w/v) dextrose for 12 hr, then inoculated into a 96-well plate with 150 μL CSM-His plus 1% (w/v) D-galactose, 1% (w/v) D-raffinose, and 0.1% (w/v) BSA at 5 3 10^5^ to 10^6^ cells/mL, and shaken at 30°C for 7 h. Every plate had biological triplicates of *Sc*Ste6 and *Yl*Ste6 populations as controls to normalize the autocrine signal for each clone (described below). Plasmids were extracted from clones that showed a significant increase in the autocrine signal (relative to *Yl*Ste6 expressing cells) (Figure S4). The samples were either transformed into chemically competent *E. coli* (DH5*α*), for retransformation into fresh autocrine strains (ySS491), or used as template for PCR amplification of TM4–6 or TM10–12 regions for Sanger sequencing. From the isolated plasmids, 49 clones were retransformed from first two TM4–6 libraries and first TM10–12 library, and biological triplicates of these selected clones were tested in the autocrine assay. Retransforming the selected clones revealed that the autocrine signal of clones from the initial selection was a good indicator of transporter function, and physiological variation or mutations outside the regions of *Yl*Ste6 that were subjected to PCR mutagenesis were not a source of error (Figure S5).

Because the autocrine signal of isolated clones is a good predictor of transporter function, we changed our approach, for the remaining one TM4–6 library and two TM10–12 libraries, to PCR amplify the transporter ORF (oSS04_206 / oSS04_188) from the plasmid extracts from yeast clones instead of isolating plasmids by transforming these extracts into *E. coli*. The PCR reactions were Sanger sequenced with a primer for either TM4–6 (oSS10_080) or TM10–12 (oSS10_113), depending on the source library. Sequencing chromatograms were segregated based on the source library and aligned to the WT *Yl*Ste6 sequence. These alignments were processed using custom scripts (Python 3.6 with BioPython package, provided at https://github.com/sriramsrikant/) to identify unique clones and calculate the number of mutations per position across unique clones. Next-generation Illumina sequencing was not used because the mutated region (roughly 480 bp) is much larger than a standard paired-end read. Illumina sequencing would thus have given us statistics on the number of distinct mutations per position but would not have provided reliable information on which mutations were linked to each other in individual clones. Given that we found 92 unique clones among the 243 analyzed by Sanger sequencing, we concluded that next-generation sequencing would not add significantly to our inferences.

#### Y. lipolytica semiquantitative mating assay

The *Ylste6Δ MAT*A strain (yaliSS005) was constructed from our WT *MAT*A strain (ML16507) using a chemical transformation protocol like the one used for *S. cerevisiae* [83]. We modified a quantitative mating protocol used in [84] to test the mating efficiency of strains containing various transporters, carried on the *CEN*/*URA3Yl* plasmid, against our *MAT*B partner (ML16510). Briefly, exponentially growing cultures in YPD of the plasmid-transformed strain and its mating partner were harvested and 2.5 × 10^6^ cells of each partner were mixed in 150 μL sterile water + 0.02% (w/v) BSA. Mating mixtures were transferred onto filters (0.22 μm pore hydrophilic PVDF 25 mm membrane, Millipore Sigma Ref: GVWP02500) using a filter assembly (with the cells spreading to about 5 mm radius), and the filters (with cells) were moved onto YM mating media plates (3 g/L yeast extract, 5 g/L Bacto-peptone, 5 g/L malt extract and 20 g/L Bacto-agar) [84]. These plates were incubated at 28°C in the dark for three days (70–74 hr). After 3 days, the filters with the mating mixtures were moved into 3 mL YP plus 2% (v/v) glycerol and 0.5% (w/v) dextrose and incubated on a roller drum at 30°C for 3 h. The cultures were transferred to microfuge tubes and sonicated to disrupt clumps, before using a Coulter counter to measure the cell density. Cells were pelleted, resuspended in water plus 0.02% (w/v) BSA and 2 × 10^7^ and 5 × 10^6^ cells were plated on diploid selective media (CSM-Lys-Ade). The mating efficiency was calculated as the number of diploid cells for the experimental samples relative to the number of diploid cells from the control mating (*Yl*Ste6 expressing plasmid in *MAT*A + *MAT*B; average set to 1) performed in the same experiment (code on Github at https://github.com/sriramsrikant/). The experiment was repeated 4 times each with biological replicates and plating replicates to account for the intrinsic noise of mating mixtures. Expression of transporters was estimated by flow-cytometry analysis of the ymKate2-tagged transporter, as the median of populations corrected for the background fluorescence of strains that lacked a fluorescently tagged transporter.

#### Sequence alignments and building homology models of YlSte6

The HMMER algorithm [65] was used to identify homologs of Ste6 from a database of fungal proteins curated and maintained by Dr. Jim Thomas (U. of Washington). Roughly 24,000 homologous sequences were identified, down-sampled to sequences that are less than 90% identical, aligned with “hmmalign” and a sequence similarity tree constructed with “FastTree” [85]. We used the clade in the sequence tree that contained the known pheromone exporters and excluded known paralogous sequences. These sequences were aligned with “hmmalign,” filtered with “hhfilter” to highlight positions present in *Yl*Ste6 and sequences that have large gaps were eliminated. The remaining 1126 transporters were considered orthologous pheromone exporters and the alignment is provided as Mendeley Source Data File 1.

Homology models were built using the core transporter sequence of *Bos taurus Bt*MRP1 (residue 314–1516) from the Leukotriene C4-bound (substrate-bound) inward-open (PDB: 5UJA) and ATP-bound outward-open (PDB: 6BHU) structures (Mendeley Source Data Files 2 and 3). *Bt*MRP1 structures were chosen because the same transporter has been resolved in both the substrate-bound, inward-open and outward-open conformations. *Bt*MRP1has a 19% sequence identity (29% sequence similarity) with *Yl*Ste6 and 18% sequence identity (29% sequence similarity) with *Sc*Ste6. The structures were submitted to SWISS-MODEL [58, 59] using the *Yl*Ste6 sequence as the query and the PyMOL Molecular Graphics System (version 2.2.3) [60] was used to plot the electrostatic surface of each model with the APBS plugin. The surface region colored white highlights the hydrophobic band of the structure that corresponds to the transmembrane region, suggesting that the overall model is correct (Figure S1C), although there could be errors in the registry of helices. The curated alignment of 1126 pheromone transporters was run through the ConSurf server [61, 62] to identify and plot the conservation at each position and plotted on the inward-open and outward-open homology models (Figure S1D). The enrichment of conserved positions in structural contacts and protein core further support the validity of our structural models.

### QUANTIFICATION AND STATISTICAL ANALYSIS

All quantitative analysis and statistical tests were performed in Python. Flow cytometry data was imported into Python via the FlowCytometryTools package (https://pypi.org/project/FlowCytometryTools/) for analysis. Sequence analysis was done in Python using the BioPython package (https://pypi.org/project/biopython/). Chi-square (*X*^*2*^) goodness-of-fit tests were performed on data in Figures 3C and S3E with the mean estimated Poisson distribution as the null model. The statistical tests and replicate details for experiments have been described in the corresponding figure legends.

### DATA AND CODE AVAILABILITY

The code generated during this study is available at Github in the repository https://github.com/sriramsrikant/2020-CB_ABC-selectivity. Code to analyze different experiments are provided as separate Jupyter lab notebooks (.ipynb). The data that support the findings of this study are available from the Lead Contact upon request. The authors declare that all data reported in this study are available within the paper, its supplementary information files and a dataset containing protein alignment, homology models, and raw autocrine data uploaded to Mendeley Data (https://doi.org/10.17632/rp3hnvh494.1).

## Supplementary Material

1

## Figures and Tables

**Table:** KEY RESOURCES TABLE

REAGENT or RESOURCE	SOURCE	IDENTIFIER
Chemicals, Peptides, and Recombinant Proteins		
*S. cerevisiae α*-factor	Bio-Synthesis	WHWLQLKPGQPMY (custom peptide synthesis)
G418	Thermo Fisher	Cat#11811031
Cycloheximide	Millipore Sigma	Cat#C7698
Deposited Data		
Selecting for altered substrate specificity reveals the evolutionary flexibility of ATP-binding cassette transporters, Srikant S et al.	Mendeley Dataset	https://doi.org/10.17632/rp3hnvh494.1
Experimental Models: Organisms/Strains		
Yeast strains	See Table S2	N/A
Oligonucleotides		
Primers	See Table S4	Custom oligo synthesis
Software and Algorithms		
SWISS-MODEL	[58, 59]	https://swissmodel.expasy.org/
PyMol Molecular Graphics System	[60]	https://pymol.org/2/
Consurf	[61, 62]	https://consurf.tau.ac.il/
Fiji	[63, 64]	https://imagej.net/Fiji
Analysis scripts	https://github.com/sriramsrikant/2020-CB_ABC-selectivity	Python scripts
HMMER	[65]	http://hmmer.org/
Other		
Atomizer	Oenophilia	Cat#900432

## Highlights

- Fungal pheromone export system offers testing ground forABC transporter evolution
- Many mutations dispersed over the transmembrane domain alter substrate selectivity
- Co-selected mutations have an at least additive effect of on substrate transport
- ABC exporters have an easily traversed substrate selectivity evolutionary landscape
