# Supplementary material for: Selecting for Altered Substrate Specificity Reveals the Evolutionary Flexibilityof ATP-Binding Cassette Transporters
Source: Curr Biol. Author manuscript; Available in PMC 2021 May 4. (PMC7243462; doi:10.1016/j.cub.2020.02.077)
Supplement: 1 [file NIHMS1572001-supplement-1.pdf]

Current Biology, Volume *30*

Supplemental Information

Selecting for Altered Substrate Specificity Reveals the Evolutionary Flexibility of ATP-Binding Cassette Transporters

Sriram Srikant, Rachelle Gaudet, and Andrew W. Murray

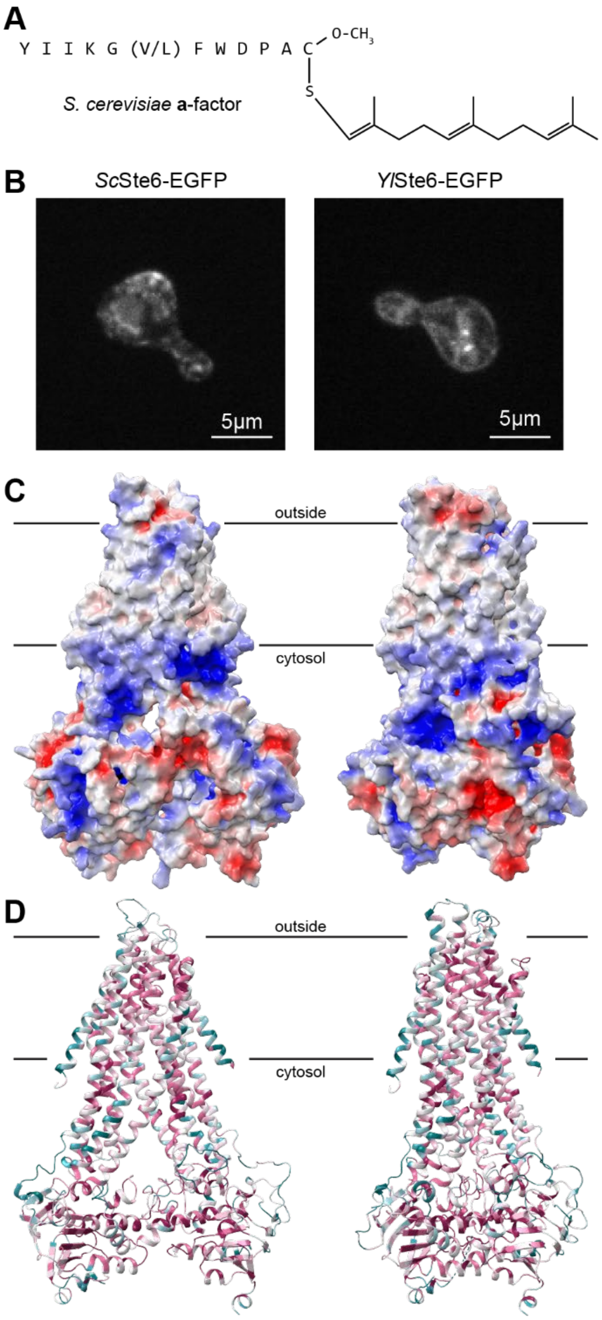

Figure S1. Lipidated pheromone export by type I ABC exporters is the first step in fungal mating. Related to Figure 1. (**A**) Mature **a**-factor from *S. cerevisiae* is produced from two genomic loci, *MFA1* and *MFA2*, which differ from each other by either encoding valine (V) or leucine (L) at position 6 in the mature pheromone. The maturation involves S-farnesylation of the C-terminal cysteine and methyl esterification of the C-terminal carboxyl moiety. (**B**) Fluorescent images of *MAT**a*** cells expressing *Sc*Ste6 and *Yl*Ste6 C-terminally tagged with EGFP to show localization to the plasma membrane and internal membranes. Images captured by spinning-disk confocal microscopy with 488 nm laser excitation. (C, D) Validation of the *Yl*Ste6 homology models. The views are the same as that used throughout the figures in the paper. (**C**) Electrostatics surface representations of the inward-open (left) and outward-open (right) homology models of *Yl*Ste6 we generated for this study using SWISS-MODEL are consistent with expectations for the membrane-embedded ABC transporter. The surfaces are colored from red (-5 kT) to blue (+5 kT). The electrostatics were generated in PyMOL using the APBS plug-in at 150 mM osmolarity. (D) Inward-open (left) and outward-open (right) models colored by conservation scores calculated by the ConSurf web server with conserved positions colored magenta and variable positions colored cyan. The curated alignment of 1126 fungal pheromone exporters was provided to ConSurf to calculate conservation scores (see STAR Methods).

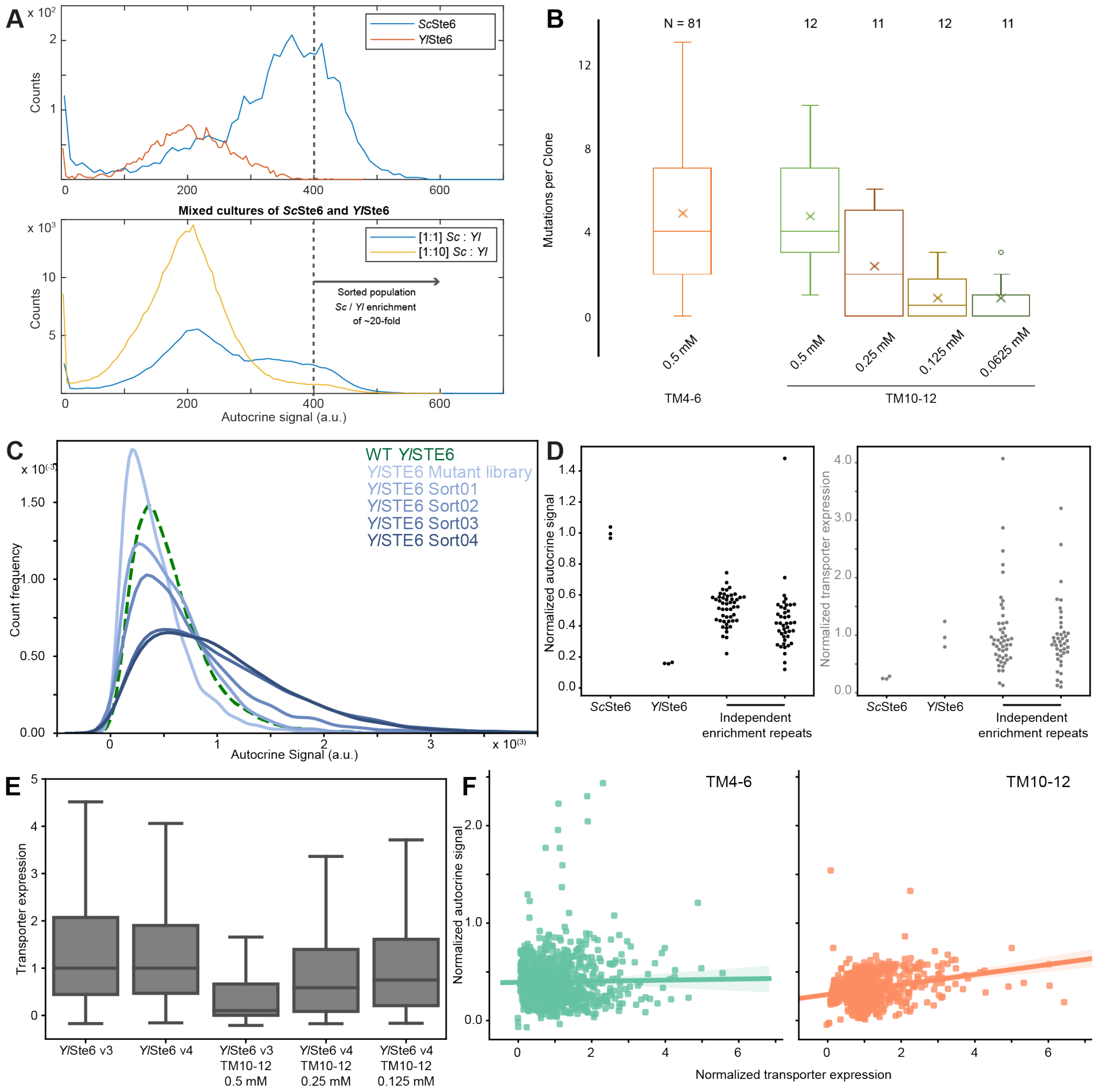

Figure S2. Autocrine system can select for increased *Sc*a-factor export by type I ABC exporter. Details of mutant library construction and enrichment for autocrine signal related to Figure 2. (**A**) Histograms of flow cytometry events in isolated *Sc*Ste6 or *Yl*Ste6 populations set a sorting gate on autocrine signal (~1% *Yl*Ste6 population) such that we can effectively enrich a 1:1 mixed population of *Sc*Ste6 *and Yl*Ste6 cells for *Sc*Ste6 by 20-fold. (**B**) Box-whisker plot of mutations per clone based on alignment of sequenced unselected clones with the corresponding regions of WT *Yl*Ste6, with mutations counted as nucleotide substitutions. The plot contains 82 clones for 0.5 mM Mn^{2+}-mutagenized TM4-6 library and 12, 11, 12 and 11 clones for 0.5 mM, 0.25 mM, 0.125 mM and 0.0625 mM Mn^{2+}-mutagenized TM10-12 libraries respectively. Means are plotted as crosses (X) within each distribution with outliers plotted as open circles. Box corresponds to middle quartiles and whiskers to outer quartiles. (**C**) Distributions of autocrine signal over four rounds of FACS-based enrichment for a representative library, highlighting the selection for cells with a right shift in the distribution of the autocrine signal. The dashed green line represents a population of cells expressing WT *Yl*Ste6, while the progressively darker solid lines represent the library through rounds of FACS enrichment. (**D**) Clones isolated from replicate

enriched populations from the same starting library were tested individually by the flow cytometry autocrine assay to measure the clone's autocrine signal (left) and transporter expression (right). Each point in a vertical group of points represents the median measurement for a given clone. (**E**) Box-whisker plot of transporter expression from flow cytometry events from unselected *YlSTE6* mutant libraries mutagenized with 0.5, 0.25, and 0.125 mM Mn^{2+}. *YlSTE6* v3 and v4 correspond to replicate populations with WT transporter measured in independent trials with different mutagenic libraries. Box corresponds to middle quartiles, horizontal black line to median, and whiskers to outer quartiles. (**F**) Scatter plot of median normalized autocrine signal and median transporter expression of all selected clones from TM4-6 (852 clones) and TM10-12 (762 clones) libraries. The linear regression of normalized autocrine signal versus transporter expression (solid lines) show little to no correlation across all clones. We speculate that the overexpression of the transporter under the *GAL1* promoter leads to saturating levels of the transporter on the plasma membrane. Thus, reducing expression does not reduce the number of transporters in the membrane and lower the rate of pheromone export.

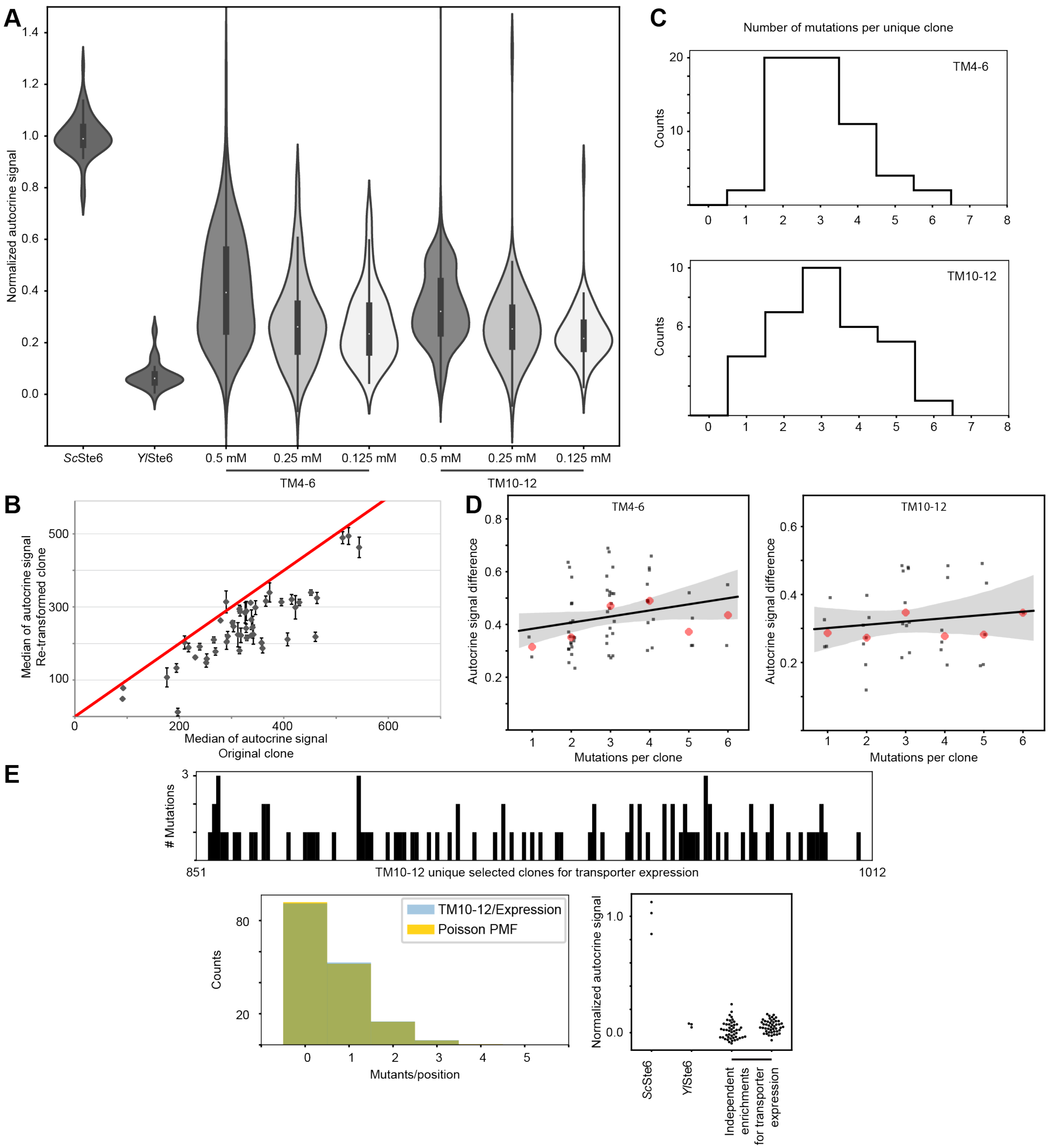

Figure S3. Selected clones show increased autocrine signal relative to WT *Yl*Ste6. Details of all clones from enrichment related to Figure 3. (**A**) The normalized autocrine signal of isolated, selected clones pooled across replicate libraries of mutated TM4-6 (852 clones) and TM10-12 (762 clones) are consistently higher than the autocrine signal of populations expressing WT *YlSTE6* (98.0% for TM4-6 and 96.9% for TM10-12 clones are higher than WT *YlSTE6*). The signals from clones isolated from libraries mutagenized with 0.5, 0.25 and 0.125 mM Mn^{2+} (shades of gray, dark to light respectively) are consistent with the fact that autocrine signal correlates with the number of (beneficial) mutations post-enrichment. See Mendeley Source Data File 5. (**B**) Plasmids were isolated from selected clones with high autocrine signal (44 clones from TM4-6 libraries and 5 clones from TM10-12 libraries) and re-transformed into the naïve autocrine strain. The autocrine signal of re-transformed selected

plasmids was measured by the flow cytometry-based autocrine assay, and error-bars (when present) represent standard deviation of biological triplicates. The autocrine signal of selected clones is a good predictor of the autocrine signal produced when the plasmids containing mutated *Yl*Ste6 are introduced into naïve strains that have not been subject to selection. (**C**) Histograms of the number of mutations per unique, selected clone from TM4-6 libraries (59 unique clones, top) and TM10-12 libraries (33 unique clones, bottom). (**D**) Relationship between the change in autocrine signal (i.e. with WT *Yl*Ste6 signal subtracted) of selected clones and the number of mutations contained with 59 clones from TM4-6 libraries (left) and 33 clones from TM10-12 libraries (right). TM4-6: Pearson coefficient 0.212, Spearman coefficient 0.273 (p_{value} 0.036); and TM10-12: Pearson coefficient 0.123 and Spearman coefficient 0.091 (p_{value} 0.613). The correlation is stronger when considering bins with at least five clones, i.e. up to four mutations per clone (Pearson coefficients 0.364 and 0.191 for TM4-6 and TM10-12, respectively). Gray dots are individual clones, red dots are medians for all the clones with a given number of mutations. (**E**) Mutations at each position summed over all 36 unique clones selected only for transporter expression are plotted in black bar plots (top). Histogram of number of mutations summed over all unique selected clones. The histogram (bottom left) of the mutations/positions (blue) is comparable to a Poisson distribution (fit to mean of observed data) of the same number of mutations (gold), and chi-square (χ^2) test statistic is 0.038 (p_{value} = 0.981) against the Poisson estimate. The autocrine signal of clones selected for transporter expression (from replicate enrichment experiments of independently constructed mutant libraries) are plotted as a swarm (black solid dots) compared to cells expressing WT *Sc*Ste6 and *Yl*Ste6 as positive and negative controls.

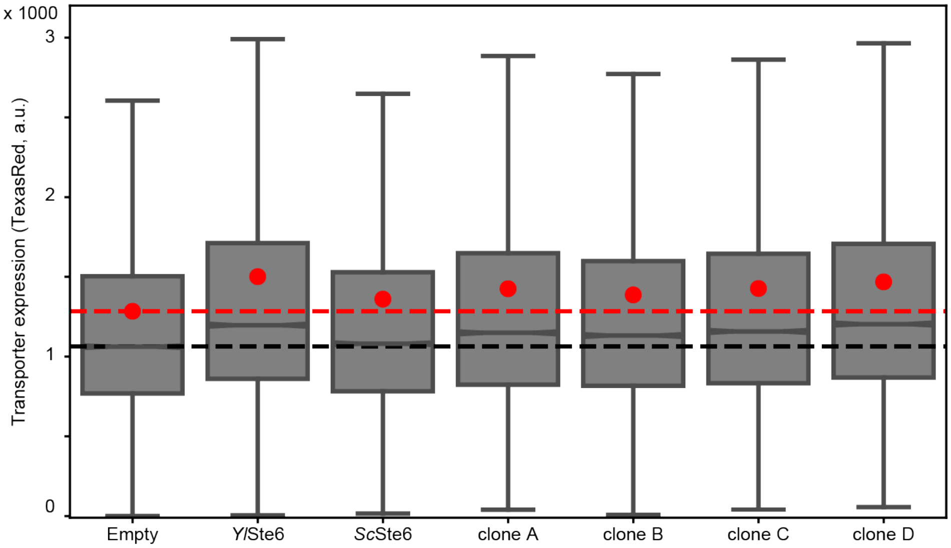

Figure S4. **Transporters are expressed in *Y. lipolytica MAT*A cells to test for mating efficiency. Related to Figure 4.** *Yl*Ste6, *Sc*Ste6, Clones A and B (TM4-6 library) and C and D (TM10-12 library) were expressed in *MAT***A** *Ylste6Δ* cells on a replicating plasmid (*CEN/YlURA3*) as used for Figure 4B. Populations were inoculated in selective medium to maintain plasmids and harvested at late exponential growth to measure fluorescently-tagged transporter expression. The transporter expression was measured from sample populations by flow cytometry (n > 20,000) and their distributions plotted as box plots with the box representing the middle 50%, and whiskers representing the outer quartiles. The central line of the boxes represents the median and the red dots represent the means of the populations. The dashed black and red lines represent the median and mean of the control population of *Y. lipolytica MAT***A** cells containing an empty vector.

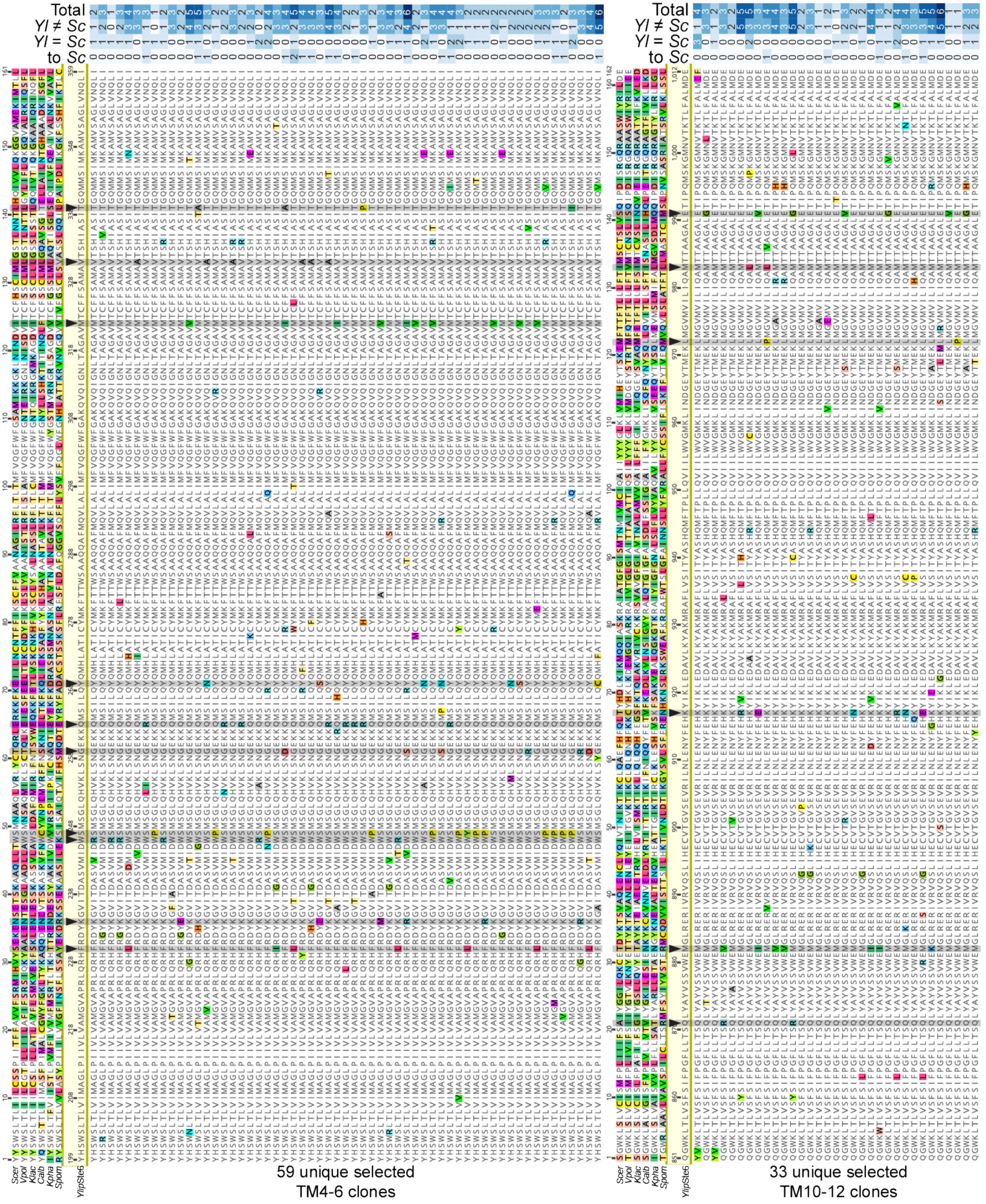

Figure S5. Mutations in selected clones are not enriched for mutations from *Yl*Ste6 to *Sc*Ste6. A listing of all the mutations in selected clones and their identity relative to WT *Yl*Ste6 and *Sc*Ste6, related to Figure 5. (**A**) Enriched plasmids from the autocrine selection of TM4-6 libraries and TM10-12 libraries were sequenced, and the translated regions of interest are aligned to pheromone transporters orthologous to *Sc*Ste6 (see STAR

Methods) with each amino acid highlighted with a distinct color at positions of non-synonymous mutations. The 59 unique clones (90 sequenced total) of TM4-6 and 33 unique clones (153 sequenced total) of TM10-12 are ordered identically to Figure 3A. The 16 identified positions that contribute to substrate selectivity are marked by arrowheads and shaded in gray.

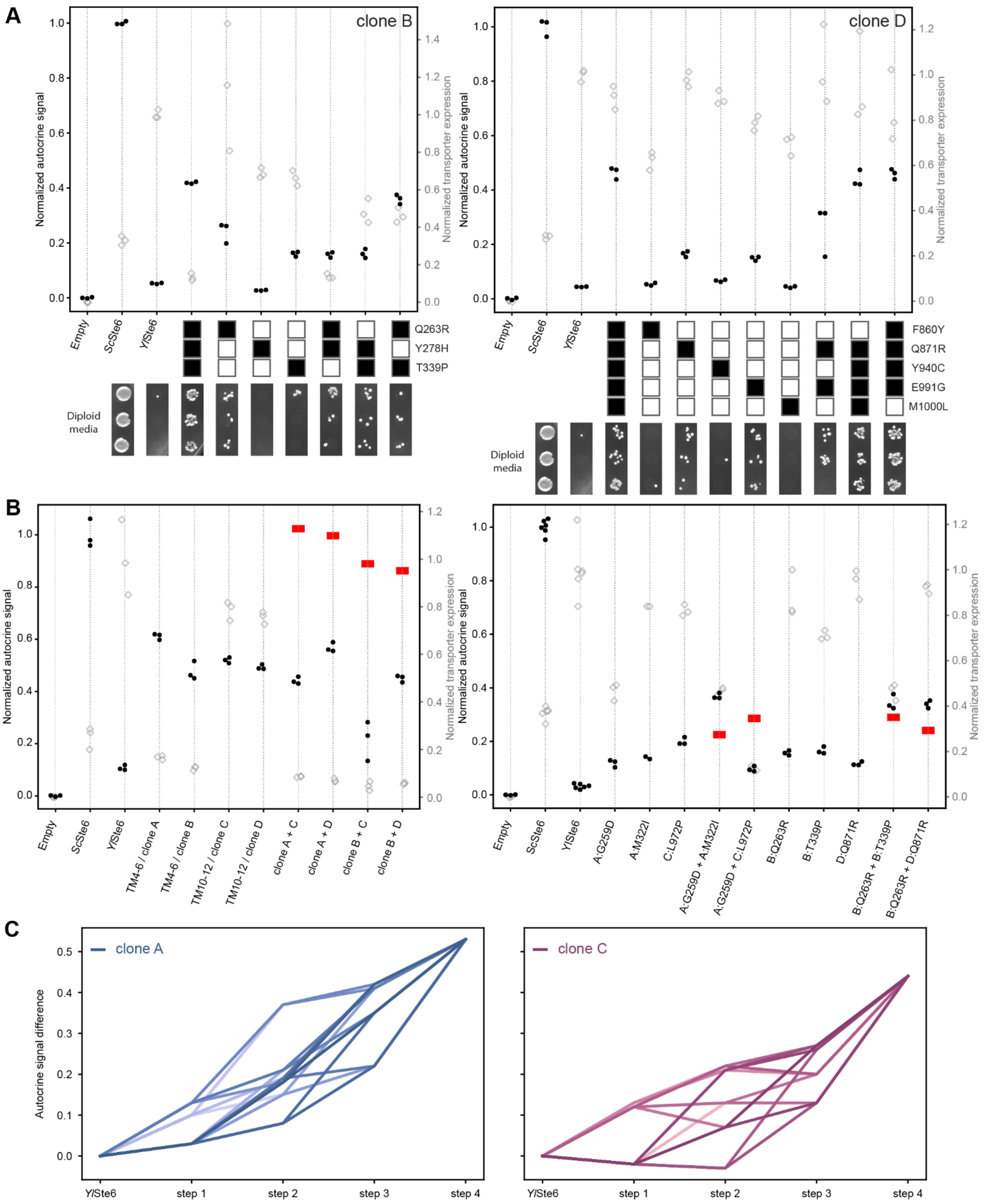

Figure S6. Mutations from selected clones have increased autocrine signal and combinations of mutations show additive autocrine signal. Autocrine signal of mutation combinations and their effect of evolutionary trajectories related to Figure 6. (**A**) Normalized autocrine signal (black solid dots) and transporter expression (gray open diamonds) of mutation combinations from clones B and D were measured using the flow cytometry

autocrine assay with biological triplicate populations (n > 25,000) for each sample. Combinations of mutations from clone B (left) and clone D (right) are represented by a series of boxes, with filled boxes representing the presence of a mutation. Mating efficiency data for cells expressing *Yl*Ste6 containing each combination of mutations are displayed below the autocrine data. Autocrine data presented here are used to plot Figure 6B. The data were collected in high-throughput autocrine experiments (see STAR Methods) to make sure samples can be consistently compared. Common samples from the left graph are duplicated in Figure 5A (top) and Figure 6A (top); and from the right graph are duplicated in Figure 4A. Mating data for *Sc*Ste6, *Yl*Ste6, clone C and clone D are reproduced from Figure 4D for comparison; all samples whose data are shown in these two figures were from the same experiment and the same *Sc*Ste6 and *Yl*Ste6 expressing controls are shown in both panels for ease of comparison. (**B**) Autocrine signal (black solid dots) and transporter expression (gray open diamonds) of chimeras of selected clones from TM4-6 libraries (clones A, B) with clones from TM10-12 libraries (clones C, D) (left) and chimeras of single beneficial mutations from selected clones (right) were measured using the flow cytometry-based autocrine assay with at least biological duplicate populations (n > 25,000) for each sample. For example, "clone A + C" is a chimera of the mutagenized regions of clones A (TM4-6) and C (TM10-12). Chimeras of single mutations from different selected clones (e.g. A:G259D + C:L972P) were compared to single mutation combinations from within the same clone (e.g. A:G259D + A:M322I). The expected autocrine signal if the effects of the mutations in both regions of the protein were additive is plotted (red bars) as a reference. (**C**) Line plots of evolutionary trajectories that go from WT *Yl*Ste6 to clone A (left, blues) and clone C (right, magentas). Having tested all possible combinations of mutations in clone A and C from Figure 6A, we can infer all possible (24) mutational trajectories. Accounting for the experimental uncertainty in the autocrine signal (0.02, as seen by data spread in Figure 6A), most trajectories climb at every step (24 from clone A and 22 of 24 from clone C), implying that most mutational paths avoid unfavorable intermediates.

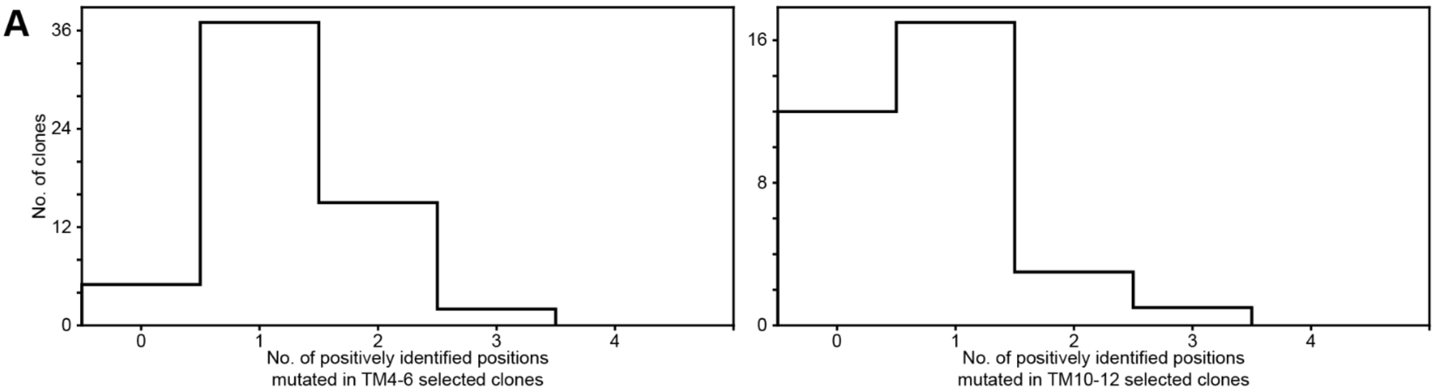

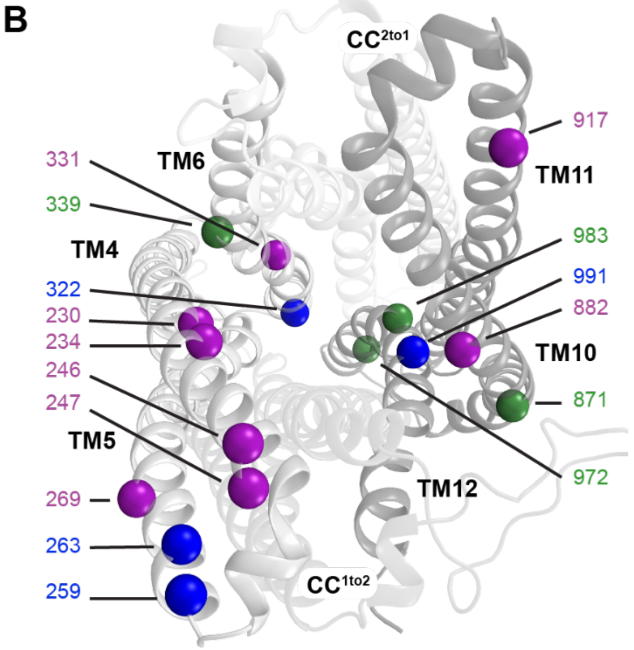

C

	Position	Residue in *Yl*	Residue in *Sc*	Mutations (counts) in all selected clones
TM4-6	230	F	S	L (6), I (1)
	234	K	N	E (2), M (1), R (3)
	246	W	W	R (6)
	247	S	S	P (12), Y (1)
	259	G	T	D (2), S (2), R (2)
	263	Q	E	R (8)
	269	Y	E	N (4), S (2), C (1)
	322	M	I	**I (3)**, V (7)
	331	V	L	A (6)
	339	T	L	A (2), P (1), I (1)
TM10-12	871	Q	A	R (2)
	882	M	T	V (3), I (3), K (1)
	917	K	T	R (2), E (2), N (2)
	972	L	M	P (2)
	983	M	I	L (2)
	991	E	S	G (4), V (3)

Figure S7. Statistical enrichment and experimental validation identify positions that affect substrate selectivity of type I pheromone exporter. Details of statistically enriched and experimentally validated positions from selected clones related to Figure 7. (**A**) Histograms of clones that contain mutations at the positions identified in Figure 7A. The histograms contain 59 clones from the TM4-6 libraries (left) and 33 clones from the TM10-12 libraries (right). (**B**) All contributing positions are highlighted as spheres on an inward-open model of the *Yl*Ste6 transmembrane domain viewed from the cytoplasm, with positions experimentally validated (green), statistically enriched (magenta) or fulfilling both criteria (blue) differentiated by color. Helices TM4-6 and TM10-12 are in light and dark gray while the rest of the TMD is shown in the lightest grays. (**C**) The details of the identified positions, showing the residues found in WT *Yl*Ste6, WT *Sc*Ste6 and mutations observed in our unique selected clones (with the number of clones with each mutation in parentheses). The only mutation to the corresponding *Sc* amino acid is shown in bold red. Positions numbers in the table are colored to indicate whether they were identified by statistical enrichment (magenta), experimental validation (green) or both criteria (blue).

Table S1. Autocrine signal difference of singly mutated transporters. Related to Figure 5.

Sample	Clone	Normalized autocrine signal	Normalized transporter expression	Autocrine signal difference[a]	Mutations per position
TM4-6 / G259D	A	0.15	0.49	0.09	6
TM4-6 / C277R	A	0.08	0.6	0.03	4
TM4-6 / M322I	A	0.19	0.94	0.13	10
TM4-6 / T339A	A	0.08	0.86	0.07	4
TM4-6 / Q263R	B	0.24	1.15	0.19	8
TM4-6 / Y278H	B	0.03	0.69	-0.03	2
TM4-6 / T339P	B	0.16	0.66	0.07	4
TM10-12 / I888V	C	0.04	0.85	-0.02	1
TM10-12 / L972P	C	0.19	0.86	0.13	2
TM10-12 / M983L	C	0.18	0.89	0.12	2
TM10-12 / A986V	C	0.04	0.83	-0.02	1
TM10-12 / F860Y	D	0.05	0.65	-0.01	2
TM10-12 / Q871R	D	0.16	0.87	0.10	2
TM10-12 / Y940C	D	0.06	0.79	0.01	2
TM10-12 / E991G	D	0.13	0.65	0.08	7
TM10-12 / M1000L	D	0.04	0.67	-0.01	1

[a] Autocrine signal difference is the difference between the normalized autocrine signal of the mutant transporter (as plotted in Figure 5A), and the mean autocrine signal of WT *Yl*Ste6 populations.

Table S2. *S. cerevisiae* and *Y. lipolytica* strains used in this study. Related to STAR Methods.

Strains

S. cerevisiae strain label	Genotype[a]	Source
SLY412	*MAT***a***; ade2-1; can1-100; leu2-3,112; his3-11,15; ura3-1; trp1-1; bud4-W303*	Murray Lab
ySS213[b]	*SLY412; BUD4; bar1Δ:TRP1Sc; ste6Δ:* P_{GAL1}*-ScerSTE6[T613A, S623A]-EGFP-*T_{STE6}*::KanMX*	This study
ySS215	*SLY412; BUD4; bar1Δ:TRP1Sc; ste6Δ:* P_{GAL1}*-KpolSTE6-EGFP-*T_{STE6}*::KanMX*	This study
ySS217	*SLY412; BUD4; bar1Δ:TRP1Sc; ste6Δ:* P_{GAL1}*-SpomSTE6-EGFP-*T_{STE6}*::KanMX*	This study
ySS221	*SLY412; BUD4; bar1Δ:TRP1Sc; ste6Δ:* P_{GAL1}*-CalbSTE6-EGFP-*T_{STE6}*::KanMX*	This study
ySS223	*SLY412; BUD4; bar1Δ:TRP1Sc; ste6Δ:* P_{GAL1}*-KlacSTE6-EGFP-*T_{STE6}*::KanMX*	This study
ySS225	*SLY412; BUD4; bar1Δ:TRP1Sc; ste6Δ:* P_{GAL1}*-KpasSTE6-EGFP-*T_{STE6}*::KanMX*	This study
ySS253	*SLY412; BUD4; bar1Δ:TRP1Sc; ste6Δ:* P_{GAL1}*-YlipSTE6-EGFP-*T_{STE6}*::KanMX*	This study
ySS393	*SLY412; BUD4; bar1Δ:TRP1Sc; ste6Δ*	This study
ySS405	*SLY412; BUD4; ste6Δ:URA3Sc*	This study
ySS491	*SLY412; BUD4; lys2Δ:*P_{FUS1}*-ymNeonGreen-*T_{FUS1}*; ste2Δ:*P_{GAL1}*-STE3; gal3:*P_{ACT1}*-GAL3-*T_{GAL3}*; ste6Δ; gal1Δ; gal10Δ:*P_{ACT1}*-reverse-tetR-cLambda-VP16-*T_{GAL10}	This study
yJHK363	*MATα; BUD4; can1-100; leu2-3,112; his3-11,15; ura3Δ*	Gift from John Koschwanez (Murray Lab)
ySS209	*yJHK363; sst2Δ:NatMX; lys2Δ:*P_{FUS1}*-ymCherry-*T_{FUS1}	This study
ySS319	*yJHK363; lys2Δ:*P_{FUS1}*-ymNeonGreen-*T_{FUS1}	This study
Y. lipolytica strain label	Genotype[c]	
ML16507	*MAT***A***; ura3-302; leu2-270; lys1-11; Ku70Δ*	Gift from Joshua Truehart (DSM ltd.)
ML16510	*MAT***B***; ura2-6861; leu2-3; ade1; Ku70Δ*	Gift from Joshua Truehart (DSM ltd.)
yaliSS005	ML16507; *Ylste6Δ:LEU2Yl*	This study

[a] Orthologous pheromone transporters are labeled as *STE6* with a string representing the species (e.g. *ScerSTE6*) and their accession details are in Table S3.

[b] Mutations [T613A, S623A] were made in *Sc*Ste6 to improve the lifetime of the protein.

[c] In *Y. lipolytica*, *MAT***A** and *MAT***B** mating types are equivalent to *MAT***a** and *MAT*α of *S. cerevisiae* respectively.

Table S3. Pheromone transporter orthologs used in this study. Related to STAR Methods.

Transporter label	NCBI ID	Species
ScerSTE6	*STE6* / NP_012713.1	*Saccharomyces cerevisiae*
VpolSTE6	Kpol1018p185 / XP_001647503.1	*Vanderwaltozyma polyspora*
KlacSTE6	KLLA0B14256 / XP_452165.1	*Kluyveromyces lactis*
CalbSTE6	*HST6* / KGR16750.1	*Candida albicans*
KpasSTE6	PAS_chr3_0858 / XP_002493091.1	*Komagataella phaffii*
YlipSTE6	YALI0E05973 / XP_503609.1	*Yarrowia lipolytica*
SpomSTE6	*MAM1* / NP_001018819.2	*Schizosaccharomyces pombe*

Table S4. Primers used to generate error-prone PCR. Related to STAR Methods.

Primer	Sequence	Purpose
oSS10_062	ACTCACTATCGCCATCTCTATGGGAGTTGCTATGTACCAT	TM4-6 5' primer for error-prone PCR
oSS10_063	ATTGTCTAACATCTACCTTGCTGGGCTTGGCGCTGTTGAT	TM4-6 3' primer for error-prone PCR
oSS10_089	CATATGCGTCGTTGGTTTCATCTGGTCGCTGGTTCAGGGC	TM10-12 5' primer for error-prone PCR
oSS10_090	CGGTTCCTCCATCCTCAGAAGTCTCTGCACGACCCTCGTC	TM10-12 3' primer for error-prone PCR
oSS10_056	GGAGTTGCTATGtacgtaTCGTGGAGTTTGACATTGG	TM4-6 5' primer for QuikChange PCR to insert SnaBI
oSS10_057	CTCCACGAtacgtaCATAGCAACTCCCATAGAGATGGCG	TM4-6 3' primer for QuikChange PCR to insert SnaBI
oSS10_060	CGTCAACCAGgaattcAACAGCGCCAAGCCCAGCAAGG	TM4-6 5' primer for QuikChange PCR to insert EcoRI
oSS10_061	GGCGCTGTTgaattcCTGGTTGACGAGTCCTGCAGATACC	TM4-6 3' primer for QuikChange PCR to insert EcoRI
oSS10_098	GTCGCTGGTTtacgtaTGGAAATTGACGCTTGTGTCCTTCTCCATCTTCCC	TM10-12 5' primer for QuikChange PCR to insert SnaBI
oSS10_099	GTCAATTTCCAtacgtaAACCAGCGACCAGATGAAACCAACGACGCATATGGTG	TM10-12 3' primer for QuikChange PCR to insert SnaBI
oSS10_100	TTTGCTCTGATGgaattcGGTCGTGCAGAGACTTCTGAGGATGGAGGAACCGAATGT	TM10-12 5' primer for QuikChange PCR to insert EcoRI
oSS10_101	CTCTGCACGACCgaattcCATCAGAGCAAACAGTGTCTTGGTGACGTTCATGCC	TM10-12 3' primer for QuikChange PCR to insert EcoRI
oSS10_080	TGGTACGACCACAACCAGGG	TM4-6 primer for sequencing
oSS10_113	GTCGGCTGTGACCACCTTCT	TM10-12 primer for sequencing
oSS04_206	TTTAACGTCAAGGAGAAAAAACTATACATATGAAAAACGACTACAAAAACTCGATTG	5' Cloning primer for YlSte6
oSS04_188	AGAGCCGCGCGGCACCAGTCCTGCGGCCGCATGACAAATACCACCATTCACCAGAGT	3' Cloning primer for YlSte6

NOTE: Lower case regions in primer sequence represent codons that are changed in the *Yl*Ste6 open reading frame.
